# Specific immune landscape of heatstroke distinguished from sepsis and aseptic inflammation

**DOI:** 10.7150/ijms.108212

**Published:** 2025-02-26

**Authors:** Juan Wu, Zhenzhen Cheng, Sha Yang, Qinjuan Wu, Ping Yang, Xinyi Liao, Tao Cheng, Wenxia Huang, Yuan Zhu, Zongcheng Tang, Min Yan, Rong Yao, Lei Du

**Affiliations:** 1 Department of Anesthesiology, West China Hospital, Sichuan University, Chengdu 610041, Sichuan, China.; 2 Department of Anesthesiology, Second Affiliated Hospital, Zhejiang University School of Medicine, Hangzhou 310000, Zhejiang, China.; 3 Department of Emergency Medicine, West China Hospital, Sichuan University, Chengdu 610041, Sichuan, China.; 4 Department of Anesthesiology, Cheng Du Second People's Hospital, Chengdu 610000, Sichuan, China.; 5 Department of Anesthesiology, Chongqing University Three Gorges Hospital, Chongqing 404100, China.; 6 Health Management Center, General Practice Medical Center, West China Hospital, Sichuan University, Chengdu 610041, Sichuan, China.

**Keywords:** lymphopenia, T cells exhaustion, Toll-like receptor 4

## Abstract

Heatstroke is associated with immune system disturbances, which was similar to sepsis and aseptic inflammation. This study characterized the immune landscape of heatstroke and compared it to sepsis or aseptic inflammation in order to identify heatstroke-specific characteristics. We prospectively recruited 40 patients with heatstroke as well as the same number of age- and sex-matched healthy controls, patients with sepsis, or cardiopulmonary bypass-induced aseptic inflammation. Blood from the four groups was collected to perform spectral flow cytometry, single-cell RNA sequencing and protein chip assay to compare the profiles of T cells, B cells, monocytes, and natural killer cells. In patients with heatstroke, the relative abundance of TLR4^+^ monocyte was significantly higher than in the other three groups, and activation of antigen presentation and inhibition of chemotaxis were observed in monocytes high expressing TLR4. Both heatstroke and sepsis were characterized by lymphopenia and T cell exhaustion, with T cell exhaustion in particular potentially associated with death and organ injury in heatstroke. The decreased cytotoxic activity of NK cells was also observed in heatstroke. In conclusion, our study described the immunological characteristics of heatstroke, which provided the theoretical basis for exploring the immunotherapy of heatstroke.

## Introduction

As climate change continues to increase average and extreme temperatures around the world, the incidence of heatstroke is likely to increase 1, 2. Heatstroke manifests as extreme hyperthermia (>40.5 °C) and central nervous system dysfunction and multi-organ failure 3, and 20-60% of affected individuals die, while most survivors suffer long-term neurological and cardiovascular complications 1, 3. Timely diagnosis and effective treatment are the primary measures to improve the prognosis of heatstroke patients 1. However, the management of heatstroke primarily involves symptomatic treatments such as cooling and organ protection, including brain, kidney and lung protection strategy, while there remains a notable lack of targeted therapies addressing the underlying pathogenesis of heatstroke 3, 4.

Immune system disturbances constitute a crucial pathophysiological mechanism of heatstroke. The present study pursued this goal within the “dual pathway model” 5, according to which the pathogenesis of heatstroke reflects (1) heat-induced necrosis and apoptosis of tissue 6, which releases numerous cytoplasmic materials into the circulation, which bind to Toll-like receptor 4 (TLR4) and other receptors to promote inflammatory responses 7-10, echoing the aseptic inflammatory responses caused by trauma or hemorrhage 3. The pathogenesis also reflects (2) heat-induced passage of lipopolysaccharides produced by gut flora into the circulation, which stimulates endotoxemia and inflammatory responses, which is similar to the pathogenesis of sepsis 11-13. Indeed, individuals who suffer heat exhaustion after strong exertion at high temperatures suffer nausea, vomiting and diarrhea, reflecting gastrointestinal disturbance, together with increased levels of lipopolysaccharides in plasma 14, 15. Thus, the pathogenesis of heatstroke partially converges with that of endotoxemia and aseptic inflammation, yet the specific immunological signatures distinguishing heatstroke from these conditions remain elusive.

Therefore, we hypothesize that identifying characteristic immune dysfunction in heatstroke may facilitate discovering immune signatures associated with organ injury induced by heatstroke. We compared the relative abundances and gene expression of immune cell types among four groups using multi-channel spectral flow cytometry and single-cell RNA sequencing to describe the characteristic immune landscape in heatstroke: patients with heatstroke; patients with sepsis, who represented endotoxemia; patients undergoing cardiopulmonary bypass, who represented aseptic inflammation; and healthy controls. Further, by comparing the immune characteristics of heatstroke patients who experienced versus those who did not experience death or organ injury, we aimed to discover immune signatures associated with organ injury induced by heatstroke. Our study explored the specific immunological signatures distinguishing heatstroke from endotoxemia and aseptic inflammation to advance our understanding of how heatstroke progresses and find the potential targets of immunotherapy.

## Results

### Comparison of basic characteristics and outcomes among four groups

**Figure 1:**
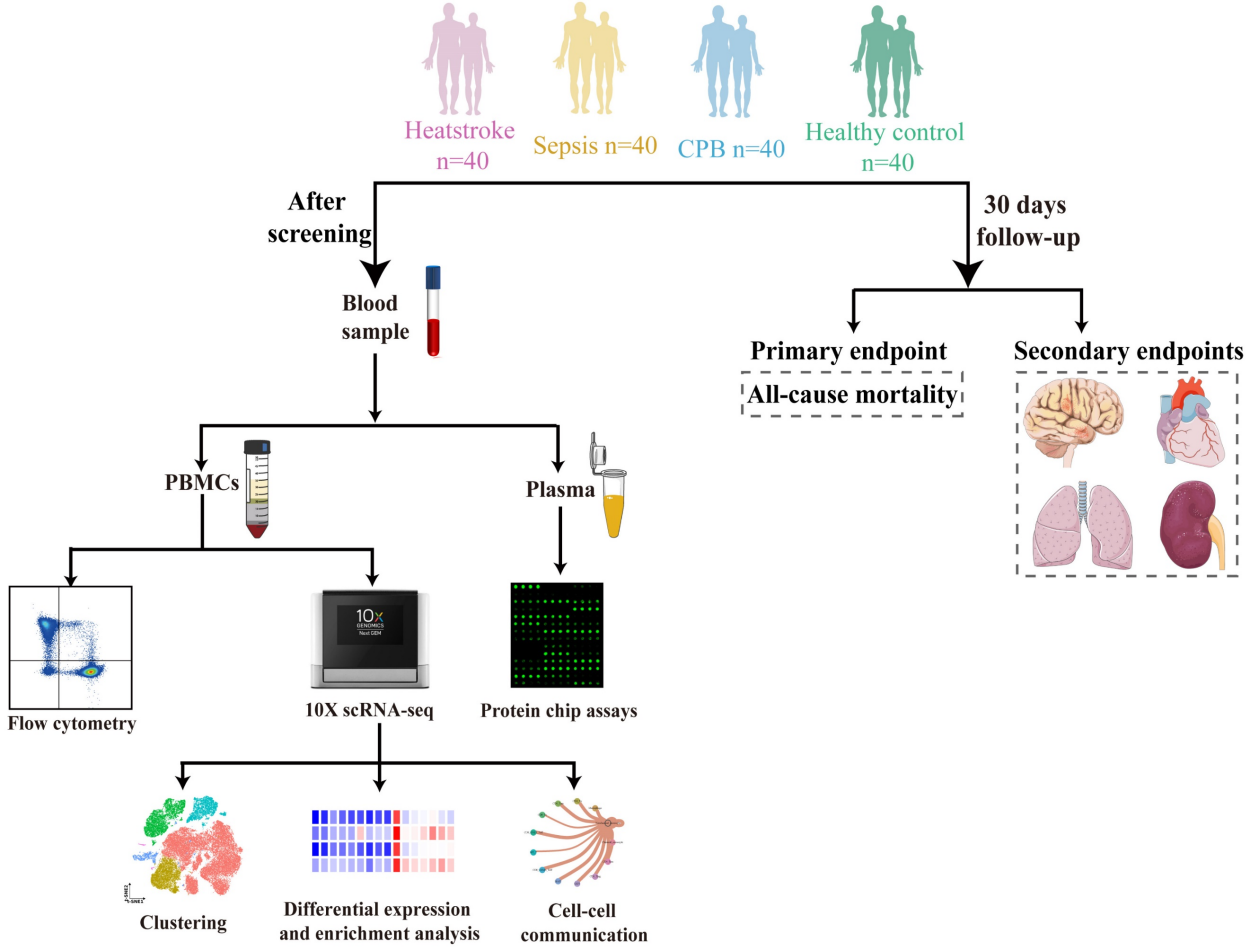
Schematic of this study. Blood was sampled from age- and sex-matched individuals who were diagnosed with heatstroke or sepsis, who underwent cardiopulmonary bypass (which induces aseptic inflammation) or who were healthy controls (40 people per group). All samples were analyzed using flow cytometry to assay relative abundances of immune cell types and subpopulations, samples from two people per group were analyzed using sc-RNA seq to examine relative abundances and their gene expression, and samples from 10 people per group were analyzed to determine levels of cytokines and chemokines in plasma. All participants were followed up for 30 days to observe all-cause mortality, nervous system dysfunction, acute heart failure, acute lung injury and acute kidney failure. CPB, cardiopulmonary bypass; PBMCs, peripheral blood mononuclear cells.

We prospectively analyzed the blood of 40 individuals in each of four groups: healthy controls and patients with heatstroke, sepsis or cardiopulmonary bypass-induced aseptic inflammation. The remaining three groups were matched to patients with heatstroke based on age and sex (**Figure 1**).

Compared to sepsis patients who were admitted an average of 60 hours after symptom onset, heatstroke patients were admitted an average of 24 hours after symptom onset (**Table 1**). Among heatstroke patients, 22 were diagnosed with exertional heatstroke, and 18 were diagnosed with classic heatstroke. Notably, exertional heatstroke patients were younger (45 years vs. 69 years, p<0.0001) and had a higher proportion of male patients (86% vs. 33%, p=0.001) than classic heatstroke patients (**Table S1**). Heatstroke patients scored a median of 9 on the Sequential Organ Failure Assessment and 7 on the Glasgow Coma Scale. Five heatstroke patients (12.5%) died within 30 days after admission, compared to 14 patients (35.0%) with sepsis (**Table 2**). Delirium affected nearly 40% of patients with heatstroke or sepsis, which was much more frequent than among patients undergoing cardiopulmonary bypass. The three groups also differed in prevalence of acute heart failure, acute lung injury and acute kidney failure.

### The specifically increased abundance of TLR4^+^ monocytes in heatstroke

**Figure 2:**
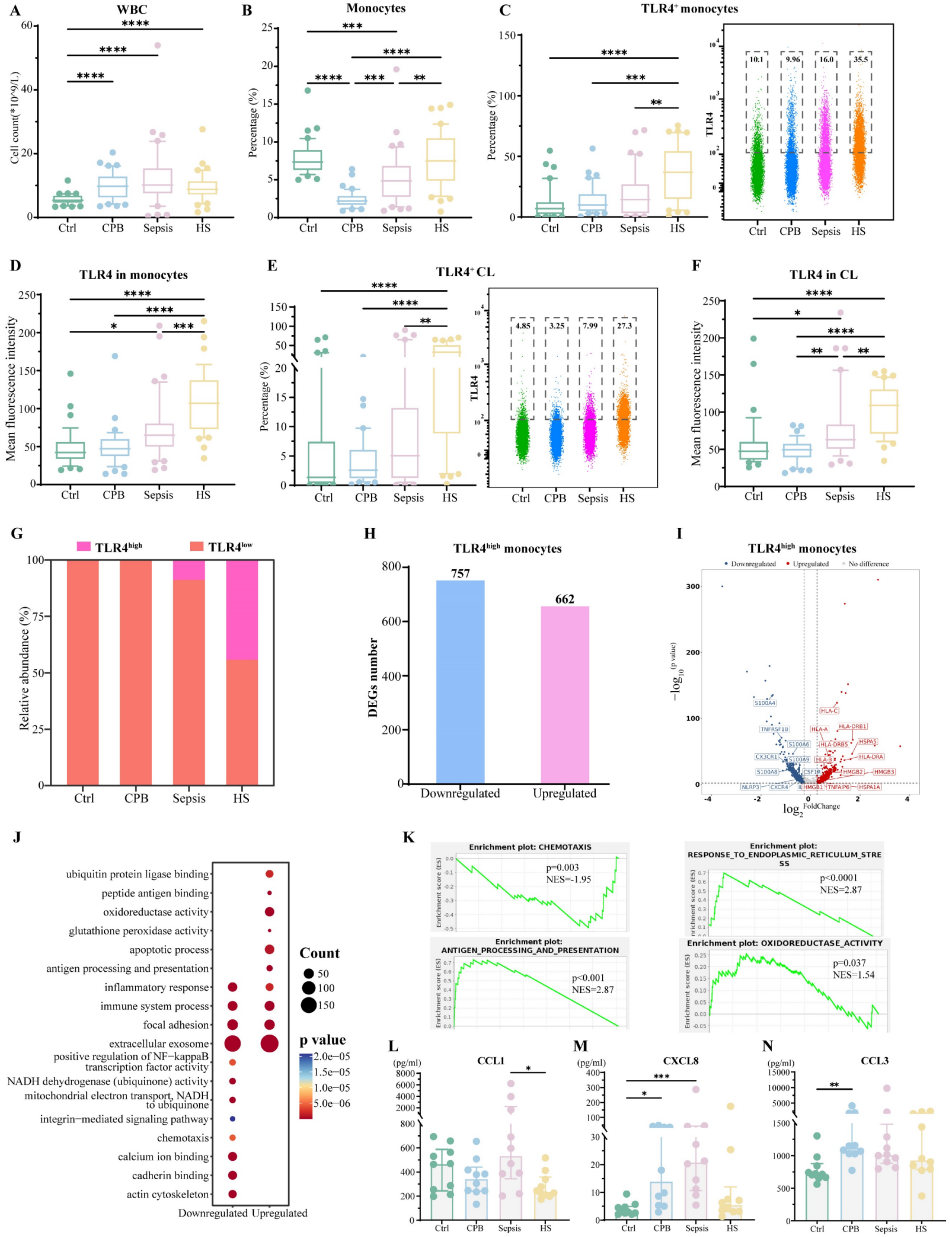
Heatstroke is associated with increased abundance of a monocyte subpopulation expressing high levels of Toll-like receptor 4 (TLR4). Samples came from the blood of healthy controls (Ctrl), patients undergoing cardiopulmonary bypass (CPB), or patients diagnosed with sepsis or heatstroke (HS). **(A-B)** The box plots of (A) white blood cells count and (B) relative abundance of monocytes among white blood cells (n=40 in each group). **(C-D)** The box plots of (C) relative abundance of TLR4-expressing cells among monocytes, and (D) mean fluorescence intensity of TLR4 on the surface of monocytes (n=40 in each group). Individual samples are shown as a scatter plot on the right, where median abundances are written above each distribution. **(E-F)** The box plots of (E) relative abundance of TLR4-expressing cells among classical monocytes, and (F) mean fluorescence intensity of TLR4 on the surface of classical monocytes (n=40 in each group). Individual samples are shown as a scatter plot on the right, in which the median abundances are written above each distribution. Significant differences among groups were assessed using Kruskal- Wallis test, *p ≤ 0.05, **p < 0.01, ***p < 0.001 and ****p < 0.0001. **(G)** Relative abundance of monocytes expressing low (TLR4^low^) or high (TLR4^high^) levels of TLR4, based on sc-RNA seq. **(H-I)** Numbers and volcano plot of heatstroke-specific differentially expressed genes in TLR4^high^ monocytes. **(J)** Enrichment of heatstroke-specific differentially expressed genes in TLR4^high^ monocytes in GO terms. **(K)** GSEA of heatstroke-specific differentially expressed genes in TLR4^high^ monocytes. **(L-N)** Levels of (L) chemokine (C-C motif) ligand (CCL1), (M) chemokine (C-X-C motif) ligand 8 (CXCL8), and (N) chemokine (C-C motif) ligand 3 (CCL3) in plasma (n=10 in each group). Median with IQR is shown. Significant differences among groups were assessed using Kruskal- Wallis test, *p ≤ 0.05, **p < 0.01 and ***p < 0.001.

While overall abundance of white blood cells in all three types of patients showed moderate increases over those in healthy controls, the proportion of monocytes was higher in patients with heatstroke than in patients with sepsis or aseptic inflammation (**Figure 2A-B, Table S2**). This increase was similarly large regardless of whether the heatstroke was related to exertion (**Table S1**).

Based on flow cytometry (**Figure S1A**), the relative abundance TLR4^+^ monocytes were significantly higher in heatstroke patients than in the three other groups (**Figure 2C**), and the mean fluorescence intensity of TLR4 on the monocyte surface was significantly higher among heatstroke patients (**Figure 2D**). The observed upregulation of TLR4 in heatstroke was also occurred on classical monocytes (**Figure 2E-F**). These results did not depend on whether the heatstroke was related to exertion (**Table S1**).

sc-RNA seq was performed to explore the gene expression profiles and function difference of immune cells. A total of 59544 cells passed the quality control threshold and were assigned to T cells, B cells, monocytes, platelets and cycling cells based on expression of canonical marker genes (**Table S3, Figure S1B-E**). Heatstroke patients were associated with significantly higher relative abundances of monocytes but significantly lower abundance of T cells (**Figure S1F-G**).

Next, we assigned monocytes to subtypes expressing high (TLR4^high^) or low (TLR4^low^) levels of TLR4 by sc-RNA seq (**Figure S2A-C**). TLR4^high^ monocytes were significantly more abundant in heatstroke patients than in the other three groups, and such monocytes were barely detectable in healthy controls or cardiopulmonary bypass patients (**Figure 2G**). The top 10 upregulated genes showing the largest differences between TLR4^high^ and TLR4^low^ monocytes were shown in **Figure S2D**. Consistently, these upregulated genes in TLR4^high^ monocytes were enriched in Gene Ontology (GO) terms related to calcium ion binding, apoptosis, cell adhesion and immune responses (**Figure S2E**).

The abundance of TLR4^high^ monocytes was too small in healthy controls and cardiopulmonary bypass patients to analyze differentially expressed genes. Comparison of gene expression in TLR4^high^ monocytes between patients with heatstroke or sepsis associated heatstroke with downregulation of 757 genes and upregulation of 662 genes (**Figure 2H, Dataset 1**). Many of the downregulated genes mediated monocyte adhesion to blood vessel walls and their migration into surrounding tissues, including the S100 genes, *CXCR4* and* CX3CR1* (**Figure 2I**). TLR4^high^ monocytes from heatstroke patients also showed downregulation of *CSF1R* and *TNFRSF1B* (also known as *TNFR2*), which encode the receptors for macrophage colony-stimulating factor (MCSF) and tumor necrosis factor (TNF)-α, as well as downregulation of the genes encoding interleukin (IL)-1β and the inflammasome component NLRP3. We confirmed substantially lower MCSF, TNFR2 and IL-1β levels in the plasma from heatstroke patients, although the differences from sepsis patients did not achieve statistical significance (**Figure S2F-H**). The downregulated DEGs were enriched in GO terms related to actin cytoskeleton and focal adhesion, while gene set enrichment analysis (GSEA) suggested the inhibition of chemotaxis and mitochondrial electron transport (**Figure 2K, Figure S2H**). Indeed, we detected lower levels of CCL1, CXCL8 and CCL3 in plasma from heatstroke patients than in plasma from sepsis patients (**Figure 2L-N**).

Many of the upregulated genes in heatstroke patients mediate antigen presentation, such as the HLA genes (**Figure 2I**). Consistently, the upregulated genes were enriched in GO terms related to antigen processing and presentation, apoptosis and oxidoreductase activity (**Figure 2J**), while GSEA indicated the activation of antigen processing and presentation, oxidoreductase activity, responses to endoplasmic reticulum stress and signaling mediated by interferon-γ (**Figure 2K**, **Figure S2H**).

These results link heatstroke to increased abundance of monocytes overexpressing TLR4 and showing enhanced antigen presentation but decreased chemotaxis and migration.

### T cell exhaustion is an undesirable feature in heatstroke

**Figure 3:**
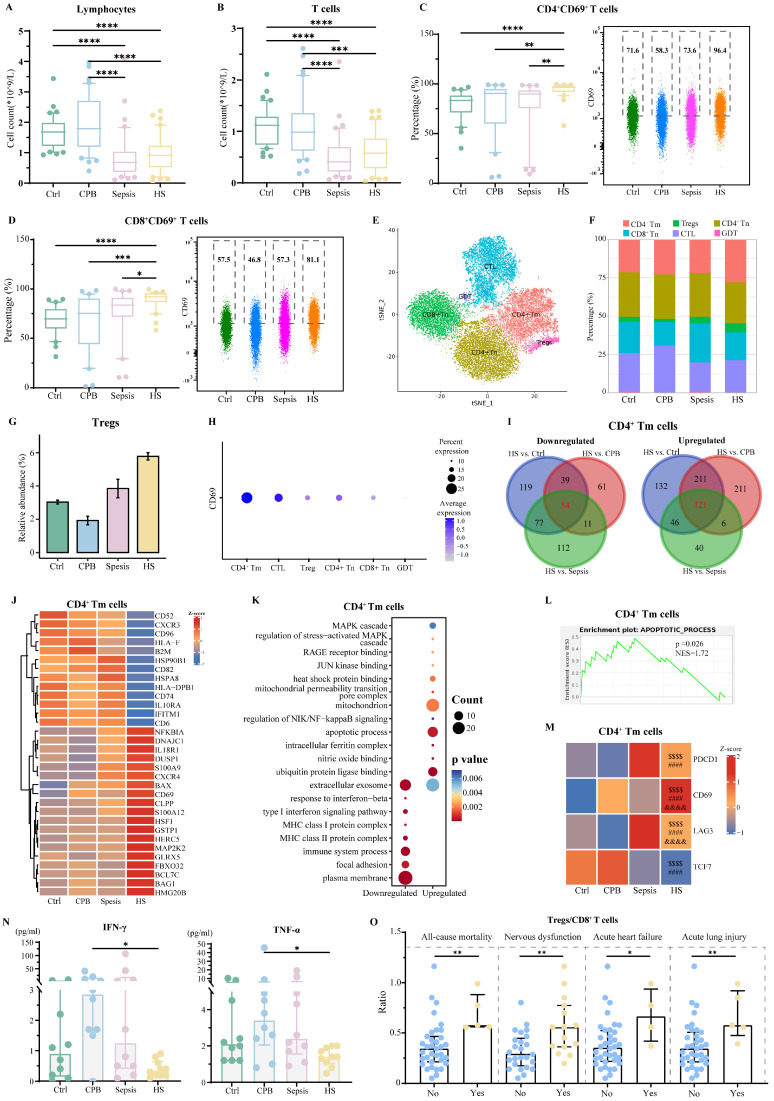
Heatstroke is associated with T cell exhaustion. Samples were those as defined in Figure 2. **(A-B)** The box plots of (A) lymphocytes counts and (B) T cells count (n=40 in each group). **(C-D)** The box plots of relative abundance of (C) CD69^+^ CD4^+^ among CD4^+^ T cells and (D) CD69^+^ CD8^+^ T cells among CD8^+^ T cells (n=40 in each group). Individual data are shown as a scatter plot on the right, where median abundances are written above each distribution. Significant differences among groups were assessed using Kruskal-Wallis test, *p ≤ 0.05, **p < 0.01, ***p < 0.001 and ****p < 0.0001. **(E)** Subpopulations of T cells identified by t-distributed stochastic neighbor embedding. Each dot represents a cell, which is colored according to subpopulation. **(F)** Relative abundance of T cell subpopulations based on sc-RNA seq. **(G)** Relative abundance of Treg cells based on sc-RNA seq. **(H)** Dot plot of CD69 expression in T cell subpopulations. **(I)** Overlap of differentially expressed genes in CD4^+^ Tm cells in three pairwise comparisons. Red numbers refer to heatstroke-specific genes. **(J-L)** Heatstroke-specific genes differentially expressed in CD4^+^ Tm cells were analyzed for (J) expression, (K) enrichment in GO terms, and (L) GSEA. **(M)** Expression heatmap of genes in CD4^+^ Tm cells related to exhaustion. $$$$, p<0.0001 in HS vs. Ctrl; ####, p<0.0001 in HS vs. CPB; &&&&, p<0.0001 in HS vs. Sepsis. **(N)** Levels of the pro-inflammatory cytokines interferon (IFN)-g and tumor necrosis factor (TNF)-a in plasma (n=10 in each group). Median with IQR is shown. Significant differences among groups were assessed using Kruskal-Wallis test, *p ≤ 0.05. **(O)** Ratios of the abundance of Treg cells to abundance of CD8^+^ T cells in heatstroke patients stratified by whether they died (No: n=35; Yes: n=5) or suffered nervous system dysfunction (No: n=25; Yes: n=15), acute heart failure (No: n=36; Yes: n=4), or acute lung injury (No: n=35; Yes: n=5) within 30 days after admission. Median with IQR is shown. Significant differences between groups were assessed using Mann-Whitney test, *p ≤ 0.05, and **p < 0.01.

Patients with heatstroke or sepsis showed similarly low relative abundances of lymphocytes and T cells, which were less abundant than in the other two groups (**Figure 3A-B**). Nevertheless, patients with heatstroke showed significantly greater expression of the activation marker CD69 on T cells than the other three groups (**Figure S3A-B**), as well as significantly higher abundances of CD69^+^CD4^+^ and CD69^+^CD8^+^ T cells (**Figure 3C-D**). These results did not depend on whether the heatstroke was related to exertion (**Table S1**).

sc-RNA seq and assignment of sequences to various subpopulations of CD4^+^ or CD8^+^ T cells based on expression of canonical markers (**Figure S3C-D**)**,** which we re-clustered in order to reduce interference from mucosa-associated invariant T cells and γδ T cells (**Figure 3E, Figure S3E-F**), indicated that regulatory T cells (Tregs, *CD4^+^ FOXP3^+^*) were significantly more abundant in patients with heatstroke than in the other three groups (**Figure 3F-G, Figure S3G**). This analysis allowed us to localize the heatstroke-induced upregulation of CD69 to CD4^+^ memory T cells (Tm, *CD4^+^CCR7^+^ANXA2^+^*) and cytotoxic T lymphocytes (CTL, *CD8A^+^ GZMK^+^/GNLY^+^*) (**Figure 3H**).

In CD4^+^ Tm cells, patients with heatstroke showed upregulation of 121 genes and downregulation of 54 genes relative to all three other groups (**Figure 3I, Dataset 2**). The upregulated genes included genes related to apoptosis and calcium ion binding (S100 genes) (**Figure 3J**), and these genes were enriched in GO terms related to apoptosis, binding of heat shock proteins and the mitochondrial permeability transition pore complex (**Figure 3K**). HSPA8 was downregulated in patients with heatstroke, which encodes HSP70, a protein responsible for attenuating apoptosis by stabilizing inhibitor of apoptosis proteins 16, 17. The downregulated genes collectively were enriched in GO terms related to type I interferon signaling and focal adhesion. GSEA indicated the activation of apoptosis (**Figure 3L**). In addition, the expression of *PDCD1* (encoding PD-1), *CD69*, and *LAG3* was higher, while expression of *TCF7* (encoding TCF1) was lower, in heatstroke patients than in healthy controls and patients with cardiopulmonary bypass, but it was not significantly different from expression in patients with sepsis (**Figure 3M**). These changes indicate T cell exhaustion in heatstroke patients 18. Indeed, flow cytometry indicated higher relative abundance of PD-1^+^CD4^+^ Tm cells and CD69^+^CD4^+^ Tm cells in patients with heatstroke than in healthy control and patients with cardiopulmonary bypass, but not in patients with sepsis (**Figure S4A-B**). Assays of plasma showed significantly lower levels of interferon-γ and TNF-α in heatstroke patients than in patients with cardiopulmonary bypass but not in patients with sepsis (**Figure 3N**), consistent with T cell exhaustion.

In CTL, patients with heatstroke showed downregulation of 120 genes and upregulation of 255 genes. One of the downregulated genes was *HSPA8*, while *PDCD1, PDCD2* and *HSPD1* were upregulated (**Figure S4C-D, Dataset 2**). Consistently, heatstroke, like sepsis, was associated with significantly larger relative abundance of PD-1^+^ cells among CD69^+^CD8^+^ T cells than in the other two groups (**Figure S4E**). Upregulated genes were enriched in GO terms related to apoptosis and binding to ubiquitin protein ligase, while downregulated genes were enriched in terms related to focal adhesion, T cell migration, and cellular response to interferon-γ (**Figure S4F**). GSEA indicated the inhibition of focal adhesion and cytokine-mediated signaling (**Figure S4G**).

Finally, we examined gene expression within Tregs because they were much more abundant in heatstroke patients. We identified 42 genes in these cells that were downregulated and 43 that were upregulated in heatstroke patients relative to the other three groups (**Figure S4H-I, Dataset 2**). One of the upregulated genes was *HSPD1*, while downregulated genes included *TGFβ1, IL10RA* and *HSPA8*. Downregulated genes were enriched in GO terms related to interferon-γ signaling, while upregulated genes were enriched in apoptosis and binding to ubiquitin protein ligase (**Figure S4J**). Compared to healthy controls, patients with heatstroke showed increases in the relative abundance of PD-1^+^ Tregs (**Figure S4K**) and the ratio of Treg cells to CD8^+^ T cells (**Figure S4L**), consistent with T cell exhaustion 19. The ratio of Treg cells to CD8^+^ T cells, a feature of T cell exhaustion, was higher among patients who died or showed nervous system dysfunction, acute heart failure or acute lung injury within 30 days after admission (**Figure 3O**), which was not observed in patients with sepsis (**Figure S4M**), possibly because the fact that the median time from symptom onset to assessment was 60 h in our study, whereas the immunosuppression that contributes to organ injury after sepsis appears to begin at least 3 days after admission 20, 21.

### Immunosuppressive B cells in NK cells

**Figure 4:**
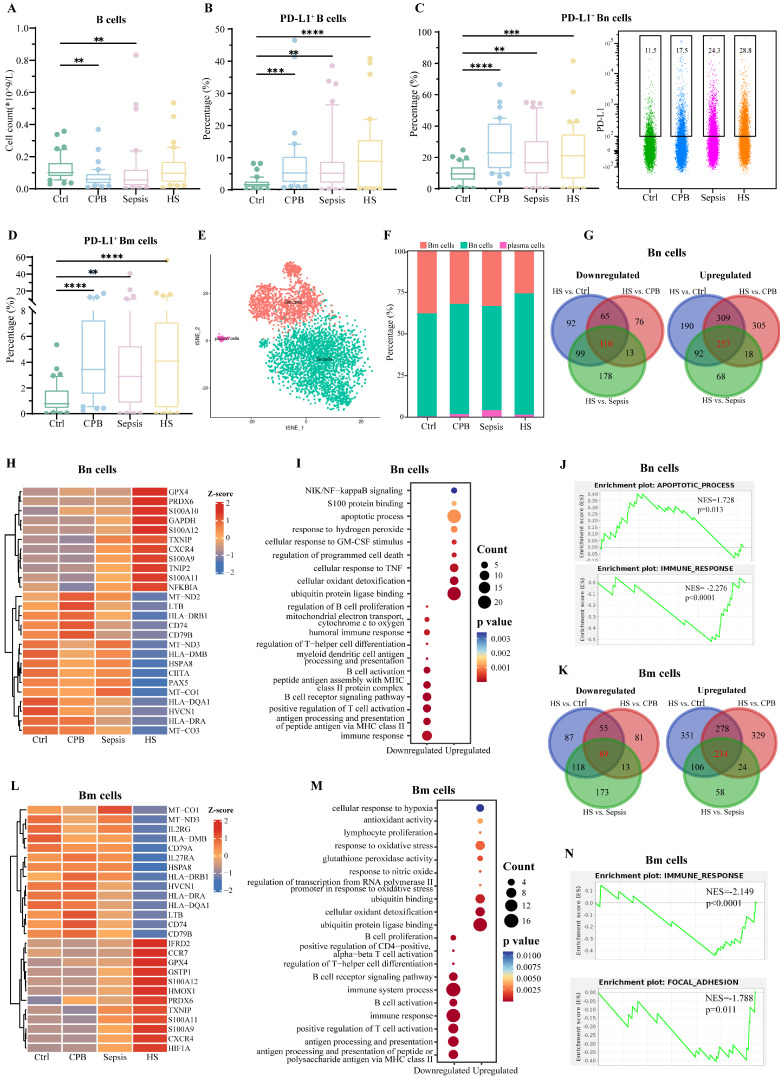
B cell signature in heatstroke. Samples were those as defined in Figure 2. **(A)** The box plots of B cells count (n=40 in each group). **(B-D)** The box plots of relative abundance of (B) PD-L1^+^ B cells among B cells, (C) PD-L1^+^ Bn cells among Bn cells and (D) PD-L1^+^ Bm cells among Bm cells (n=40 in each group). Data from single PD-L1^+^ Bn cells are shown as a scatter plot on the right, where median abundances are written above each distribution. Significant differences among groups were assessed using Kruskal-Wallis test, **p < 0.01, ***p < 0.001 and ****p < 0.0001. **(E)** Subpopulations of B cells identified by t-distributed stochastic neighbor embedding. Each dot represents a cell, which is colored according to subpopulation. **(F)** Relative abundance of B cell subpopulations. **(G)** Overlap of differentially expressed genes in Bn cells in three pairwise comparisons. Red numbers refer to heatstroke-specific genes. **(H-J)** Heatstroke-specific genes differentially expressed in Bn cells were analyzed for (H) expression, (I) enrichment in GO terms, and (J) GSEA. **(K)** Overlap of differentially expressed genes in Bm cells in three pairwise comparisons. Red numbers refer to heatstroke-specific genes. **(L-N)** Heatstroke-specific genes differentially expressed in Bm cells were analyzed for (L) expression, (M) enrichment in GO terms, and (N) GSEA. Bm, memory B cells; Bn, naïve B cells; CPB, cardiopulmonary bypass; HS, heatstroke; PD-L1, programmed death ligand-1

Overall relative abundance of B cells did not differ significantly between patients with heatstroke and patients in the other three groups, but the abundance of B cells expressing PD-L1 was significantly greater in the three types of patients than in healthy controls (**Figure 4A-B**), in particular the abundances of PD-L1^+^ naïve B (Bn) cells and memory B (Bm) cells (**Figure 4C-D**, **Figure S5A**), with no significant differences among the types of patients. These results did not depend on whether heatstroke was related to exertion (**Table S1**).

sc-RNA seq and assignment to B cell types based on expression of canonical markers (**Figure S5B-C**) showed that heatstroke was associated with an increase in relative abundance of Bn cells (*CD79A^+^ TCL1A^+^*) and decrease in abundance of Bm cells (*CD79A^+^AIM2^+^*) (**Figure 4E-F**). Comparison of gene expression in Bn cells between patients with heatstroke and individuals in the other three groups revealed 257 genes there were upregulated and 110 that were downregulated in heatstroke (**Figure 4G, Dataset 3**). The upregulated genes included genes related to inflammation and oxidative stress, such as S100 genes, and *GAPDH* (**Figure 4H**). Collectively, upregulated genes were enriched in GO terms related to apoptosis and binding to ubiquitin protein ligase (**Figure 4I**). Downregulated genes included *HSPA8* and HLA genes. Collectively, downregulated genes were enriched in GO terms related to immune response and B cell activation. GSEA indicated inhibition of immune responses and activation of apoptosis (**Figure 4J**).

Comparison of gene expression in Bm cells between patients with heatstroke and individuals in the other three groups revealed 89 genes that were downregulated and 234 that were upregulated in heatstroke (**Dataset 3, Figure 4K-L**). Downregulated genes included *HSPA8* and HLA genes, which were enriched in GO terms related to immune responses, and antigen processing and presentation (**Figure 4M**). Upregulated genes included the S100 genes, which were enriched in GO terms related to response to oxidative stress and binding to ubiquitin protein ligase. GSEA indicated inhibition of immune responses and focal adhesion (**Figure 4N**).

### Reduced cytotoxic activity of NK cells in heatstroke

**Figure 5:**
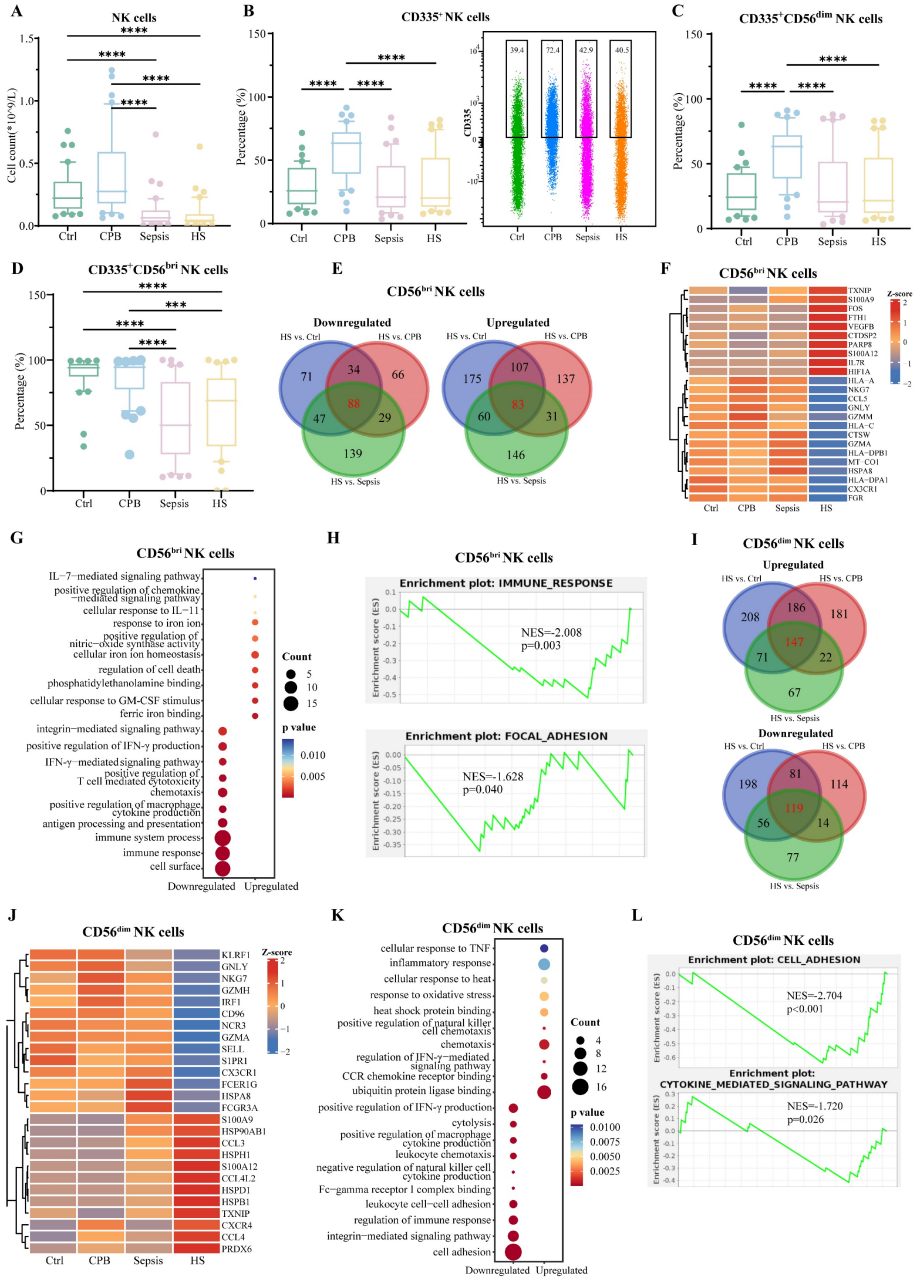
NK cells signature in heatstroke. Samples were those as defined in Figure 2. **(A)** The box plots of NK cells count (n=40 in each group). **(B-D)** The box plots of relative abundance of (B) CD335^+^ NK cells among NK cells, (C) CD335^+^CD56^dim^ NK cells among CD56^dim^ NK cells and (D) CD335^+^ CD56^bri^ NK cells among CD56^bri^ NK cells (n=40 in each group). Data from single CD335^+^ NK cells are shown as a scatter plot on the right, where median abundances are written above each distribution. Significant differences among groups were assessed using Kruskal-Wallis test, ***p < 0.001 and ****p < 0.0001. **(E)** Overlap of differentially expressed genes in CD56^bri^ NK cells in three pairwise comparisons. Red numbers refer to heatstroke-specific genes. **(F-H)** Heatstroke-specific genes differentially expressed in CD56^bri^ NK cells were analyzed for (F) expression, (G) enrichment in GO terms, and (H) GSEA. **(I)** Overlap of differentially expressed genes in CD56^dim^ NK cells in three pairwise comparisons. Red numbers refer to heatstroke-specific genes. **(J-L)** Heatstroke-specific genes differentially expressed in CD56^dim^ NK cells were analyzed for (J) expression, (K) enrichment in GO terms, and (L) GSEA. bri, bright; CPB, cardiopulmonary bypass; NK cells, NK cells; HS, heatstroke

Relative abundance of natural killer (NK) cells was significantly lower in patients with heatstroke or sepsis than in the other two groups (**Figure 5A**), and this did not depend on whether heatstroke was related to exertion (**Table S1**). The reduced abundance of NK cells reflected primarily a decrease in abundance of CD56dim (CD56^dim^) NK cells, which express chemokine receptors and kill target cells directly 22, rather than a decrease in CD56bright (CD56^bri^) NK cells, which secrete cytokines 22 (**Figure S6A-C**). Compared with cardiopulmonary bypass patients, the expression of activation marker CD335 on NK cells, CD56^dim^ and CD56^bri^ NK cells were lower in heatstroke patients (**Figure 5B-D**). These results did not depend on whether heatstroke was related to exertion (**Table S1**).

sc-RNA seq and assignment into two types of NK cells confirmed that heatstroke and sepsis were associated with lower number of NK cells than that in the other two groups (**Figure S6D-F**), rather than affecting the relative abundance of CD56^bri^ NK cells (*NKG7^+^XCL1^+^*) and CD56^dim^ NK cells (*NKG7^+^FGFBP2^+^*) (**Figure S6G**).

Comparison of gene expression in CD56^bri^ NK cells between patients with heatstroke and individuals in the other three groups identified 88 genes that were downregulated and 83 that were upregulated in heatstroke (**Figure 5E, Dataset 4**). Downregulated genes included *HSPA8*, *NKG7* and HLA genes, which were enriched in GO terms related to immune responses, and antigen processing and presentation (**Figure 5F-G**). Upregulated genes included *IL7R* and S100 genes, which were enriched in GO terms related to cellular responses to signaling mediated by interleukins-7 and -11. GSEA indicated inhibition of immune responses and focal adhesion (**Figure 5H**).

Comparison of gene expression in CD56^dim^ NK cells between patients with heatstroke and patients in the other three groups identified 119 genes that were downregulated and 147 that were upregulated in heatstroke (**Figure 5I**). Downregulated genes included *HSPA8* and were enriched in GO terms related to leukocyte chemotaxis. Upregulated genes included *HSPD1*, *CCL3, CCL4*, and *CXCR4*. Upregulated genes were enriched in GO terms related to responses to oxidative stress and binding to ubiquitin protein ligase (**Figure 5J-K**). GSEA indicated the inhibition of cell adhesion and cytokine-mediated signaling (**Figure 5L**).

### Intercellular signaling that inhibits NK cells in heatstroke

**Figure 6:**
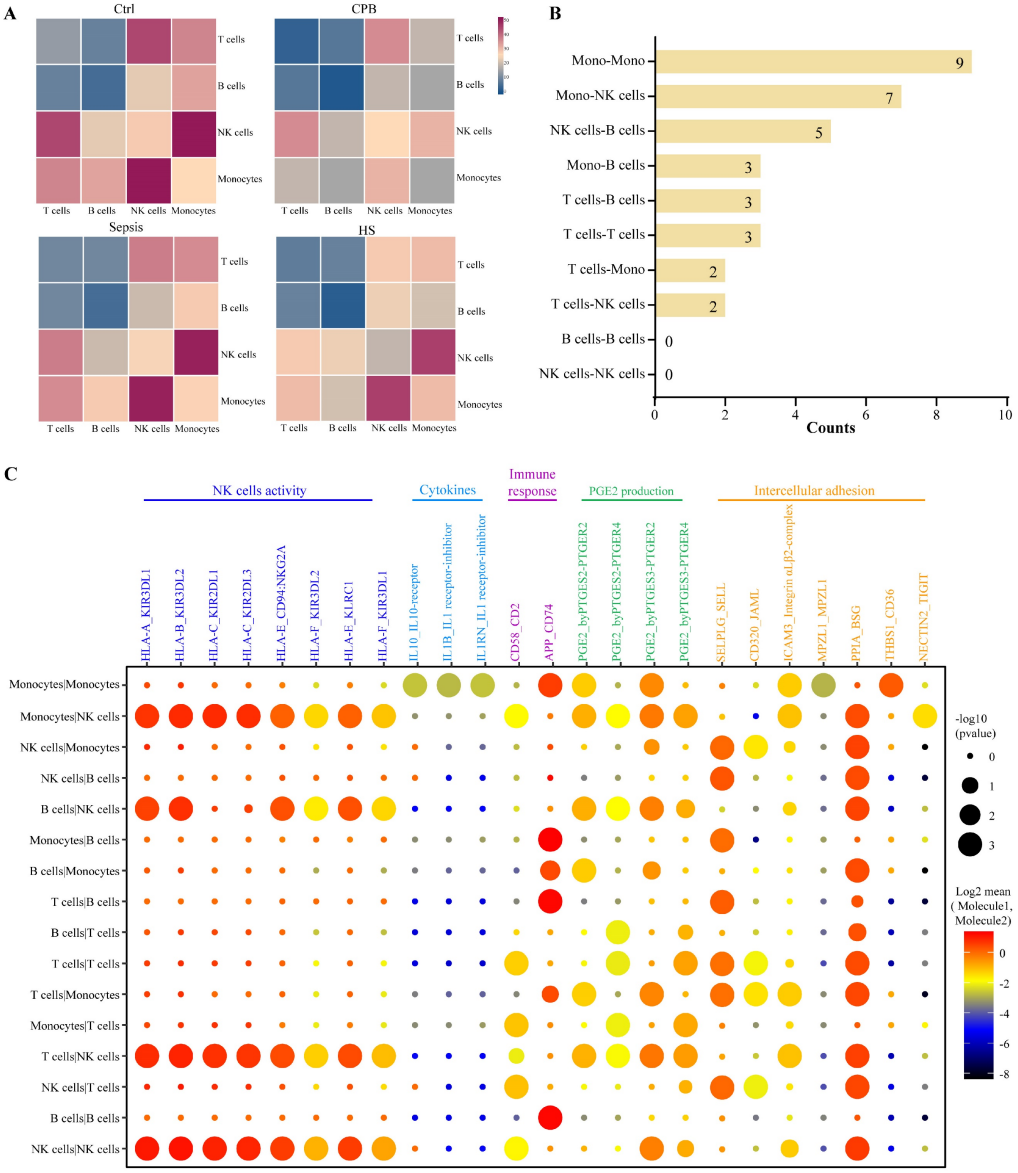
Heatstroke-specific interactions between immune cell types. **(A)** Heatmaps showing the numbers of possible interactions among T cell, B cells, monocytes and NK cells in four groups. **(B)** Numbers of heatstroke-specific interactions among T cell, B cells, monocytes and NK cells. **(C)** Dot plot of heatstroke-specific interactions, where dot size reflects the -log_10_(p value) and dot color reflects the log_2_-adjusted mean of the expression of molecule 1 in cell type 1 and expression of molecule 2 in cell type 2. NK, NK; PGE2, prostaglandin E2

Analysis of expression levels of ligands on one immune cell type and their corresponding receptors on other immune cell types identified several cell-cell interactions in patients with heatstroke that were not detected in the other three groups (**Figure 6A, Figure S7A-B**): nine interactions between monocytes, seven between monocytes and NK cells, and five between NK cells and B cells (**Figure 6B**).

These interactions were predicted to modulate the activity of NK cells, secretion of cytokines, immune responses, production of prostaglandin E2 and intercellular adhesion (**Figure 6C**). Prostaglandin E2 likely acts as a pyrogen to induce fever in patients with heatstroke. We detected interactions of the HLA ligands encoded by* HLA-A, HLA-B, HLA-C, HLA-E* and *HLA-F* on the surface of monocytes, B cells, and T cells with the immunoglobulin-like receptors encoded by *KIR3DL1, KIR3DL2, KIR2DL1, KIR2DL3*, and *CD94:NKG2A* on the surface of NK cells, and these interactions are capable of inhibiting the activation of NK cells 23, 24.

## Discussion

We provide the detailed analysis of the landscape of immune cells in blood of patients with heatstroke and the most extensive comparison of that landscape with that in the blood of patients with sepsis or aseptic inflammation. Our results identify elevated abundance of TLR4^+^ monocytes as specific to heatstroke, and further characterize monocytes with high TLR4 expression in heatstroke by their activation of antigen presentation and inhibition of chemotaxis. At the same time, we found that both heatstroke and sepsis are associated with lymphopenia and T cell exhaustion, and the degree of T cell exhaustion may be related to short-term risk of death and organ failure in heatstroke. We also found heatstroke to be associated with decreased cytotoxic activity by NK cells. Our study described the specific immunological characteristics of heatstroke and found immune signatures associated with organ damage induced by heatstroke, which provided the theoretical basis for exploring the immunotherapy of heatstroke.

Previous works have linked heat stress to an increase in the numbers of monocytes and upregulation of TLR4 5, 25-28, which binds to lipopolysaccharides (LPS) and other molecules known as “damage-associated molecular patterns” (DAMPs) that are generated during tissue injury, ultimately leading to innate immune responses and inflammatory responses 5, 26, 29. Further, we found the expression level of TLR4 on monocytes was higher than that in aseptic inflammation and sepsis. It is reasonable that expression level of TLR4 are significantly higher in patients with heatstroke than in those with aseptic inflammation, because the levels of DAMPs and LPS increase to activate TLR4 in heatstroke according to the “dual pathway model” 5, whereas LPS levels do not increase in aseptic inflammation. On the basis of the “dual pathway model”, Lim, C. L. 30 suggested that the endotoxemia pathway of heatstroke was mediated primarily by endotoxemia and systemic inflammatory response, ultimately leading to sepsis, which meant that excessive LPS leaked from gut activates TLR4 on monocytes in circulation to product pro-inflammatory cytokines 20. Further, we found the expression level of TLR4 on monocytes in heatstroke was higher than that in sepsis and could differentiate the diagnosis of heatstroke from sepsis, which wasn't found in previous study. However, in consideration of the small sample size in our study, further experiments are needed to confirm the specific increase in TLR4^+^ monocytes observed in heatstroke patients.

The effects of overexpressed TLR4 on monocytes on tissue injury induced by heatstroke were contradictory. Dehbi *et al.* 26 found that knocking out TLR4 could promote the production of IL-1β, and exhibit severer liver damage and higher mortality in the presence of extreme heat stress, which suggesting the protective role of TLR4, and we also found the downregulation of *IL-1B* in TLR4^high^ monocytes. However, many studies demonstrated the overexpression of TLR4 was associated with heatstroke-induced cardiomyocyte injury, acute lung injury and brain injury, and administrating TLR4 inhibitor, or dexmedetomidine and traditional Chinese medicine Jiawei Bai-Hu-Decoction could decrease inflammatory cytokines levels and alleviated tissue injury by inhibiting TLR4 signal pathway 31-33. In our study, the *HMGB1*, an essential mediator of TLR4 signaling and organ damage 26, was upregulated in TLR4^high^ monocytes, suggesting overexpression of TLR4 was related to organ injury in heatstroke. What's more, in sepsis or aseptic inflammation, overexpressed TLR4 was related to the increased production of CCL1 and CXCL8 in order to recruit monocytes to the sites of infection 34, which was contrary to the inhibited chemotaxis of TLR4^high^ monocytes in our patients with heatstroke. Therefore, whether the increased level of TLR4 on monocytes played a protective or destructive role in organ damage induced by heatstroke needed further exploration.

The pathological features in heatstroke that we found to be distinguished from aseptic inflammation, but similar to sepsis, were lymphopenia and immune suppression in the form of T cell exhaustion. A previous study has demonstrated that lymphocyte counts were decreased after strenuous exercise in athletes 5. Furthermore, the enrichment of ubiquitin protein ligase binding pathways in our heatstroke patients suggested that lymphocytes were targeted for degradation as heat-damaged proteins and lipids 35. What's more, the upregulation of pro-apoptotic genes *HSPD1* and *S100A9* 17, 36, 37, the downregulation of anti-apoptotic gene *HSPA8* 16, 17, and the activation of apoptosis all suggest the pathological processes underlying lymphopenia following heatstroke. These findings were consistent with previous research suggested apoptosis as the mechanisms underlying cell death induced by heat stress 38. Heatstroke patients showed T cell exhaustion that we detected as upregulation of PD-1 in T cells and its corresponding ligand PD-L1 in B cells, as well as decreases in levels of TNF-α and IFN-γ in plasma 18. We suggested that, as in sepsis, T cell exhaustion in heatstroke occurred as a compensatory response to prevent inflammatory responses from spiralling out of control but this response can end up being too effective, paralyzing necessary immune responses to tissue injury 21. Further, the degree of T cell exhaustion was related to death and organ injury caused by heatstroke in our study, and the effects of T cell exhaustion on predicting prognosis also found in patients with sepsis and certain cancers 19, 39. The upregulation of the inhibitory immune checkpoint molecules PD-1 and its corresponding ligand PD-L1 characterized immunosuppression 40, and previous studies have demonstrated that the immunomodulation strategy, via administrating anti-PD-1/PD-L1 antibodies such as nivolumab and BMS-936559, could improve survival by restoring immune cell function in sepsis models 40-42. Therefore, considering that T cell exhaustion was associated with organ damage in heatstroke patients, we suggested that future studies should explore whether blocking inhibitory immune checkpoint molecules can improve prognosis in heatstroke patients via re-activating exhausted T cells.

Reduced cytotoxic activity of NK cells in heatstroke is another formation of immune suppression to be distinguished from aseptic inflammation. NK cells kill target cells by recognizing target cells, adhering to them, and transferring lytic granules into them 43. Previous study demonstrated that heatstroke involved decreases in the number of NK cells and in their cytotoxic activity 44. Besides the reduction in the number of NK cells, our patients with heatstroke showed the downregulation of the activation marker CD335 on NK cells and the appearance of many ligand-receptor interactions inhibiting NK cell activation 23, 24, linking heatstroke to decreased NK cell activation. Furthermore, inhibition of cell adhesion may suggest the NK cells adhering to target cells was weakened, and these results comprehensively described the reduced cytotoxic activity of NK cells in heatstroke. What's more, we observed evidence of upregulated production of prostaglandin E2 in heatstroke patients, which could be induced by heat stress 45. Previous studies found that prostaglandin E2 not only contributed to fever but also inhibited cytotoxic activity of NK cells by weakening the ability of NK cells to adhere to target cells 46, 47. Therefore, we suggested that the increased production of prostaglandin E2 may be related to decreased cytotoxic activity of NK cells in heatstroke, and administrating E-prostanoid receptor antagonists may restore the proliferation and cytotoxic activity of NK cells 48.

Our results should be interpreted with caution given our small, single-center sample. Our findings should be verified and extended in larger populations. Such work should also enable the analysis of activation or exhaustion of a broader spectrum of immune cell types and subpopulations. At the same time, mechanistic studies in appropriate cell culture or animal models should verify the contributions of specific cell types and processes that we predicted here through bioinformatics and blood assays.

In conclusion, the immune landscapes in heatstroke were characterized by a significant increase in TLR4^+^ monocytes and immune suppression. The latter is manifested as lymphocytopenia and T cell exhaustion, which was associated with death and organs injury in heatstroke, and could be the immunotherapy targets in the future study.

## Materials and Methods

Materials and methods were detailed in **Supplementary materials**.

## Supplementary Material

Supplementary materials and datasets.

## Figures and Tables

**Table 1:** Demographic and clinical characteristics of the four groups of individuals in the study (n = 40 each).

Characteristic	Healthy controls	Cardiopulmonary bypass	Sepsis	Heatstroke	p
Age, yr	57 (47-68)	57 (50-66)	57 (52-67)	58 (40-69)	0.975
Sex					0.994
Male	24 (60)	25 (62.5)	25 (62.5)	25 (62.5)	
Female	16 (40)	15 (37.5)	15 (37.5)	15 (37.5)	
Body mass index, kg/m^2^	23.5 (21.9-24.2)	23.8 (22.2-25.8)	20.9 (20.0-25.8)	24.7 (22.8-26.1)	0.194
Duration from symptom onset until admission, h	NA	NA	60 (24-153)	24 (8-72)	0.001^§^
SOFA score	NA	NA	7 (4,11)	9 (6,12)	0.274^§^
Glasgow Coma Scale score	NA	NA	15 (6,15)	7 (3,11)	<0.0001^§^
**Comorbidities**					
Hypertension	2 (5)	15 (37.5)	13 (32.5)	9 (22.5)	0.004
Coronary heart disease	0 (0)	15 (37.5)	0 (0)	0 (0)	<0.0001
Chronic obstructive pulmonary disorder	0 (0)	0 (0)	1 (2.5)	0 (0)	0.389
Diabetes	0 (0)	1 (2.5)	12 (30)	4 (10)	<0.0001
**Laboratory results**					
Red blood cells, 10^12^/L	4.8 (4.5-5.4)	3.0 (2.8-3.5)	3.1 (2.3-3.7)	3.7 (2.9-4.1)	<0.0001
Hemoglobin, g/L	148 (133-155)	92 (82-104)	91 (73-108)	108 (84-124)	<0.0001
Platelets, 10^9^/L	196 (163-243)	101 (76-122)	46 (21-149)	85 (45-128)	<0.0001
White blood cells, 10^9^/L	5.4 (4.5-7.0)	9.8 (6.2-12.9)	10.2 (7.4-15.5)	8.8 (7.1-11.5)	<0.0001
Neutrophils, %	59 (52-66)	76 (69-81)	89 (79-92)	82 (72-88)	<0.0001
Lymphocytes, %	30 (24-35)	20 (16-27)	6.5 (3.5-12.7)	9.4 (5.0-14.8)	<0.0001
Monocytes, %	7.4 (6.2-9.0)	2.2 (1.6-2.9)	4.9 (2.7-6.9)	7.5 (4.8-10.5)	<0.0001

Values are n (%) or median (interquartile range). §, Significant differences between groups were assessed using Manny-whitney test; NA, not applicable; SOFA, sequential organ failure assessment.

**Table 2:** Comparison of mortality and other complications in the individuals of the study (40 per group)

Endpoint	Healthy controls	Cardiopulmonary bypass	Sepsis	Heatstroke	p
**Primary endpoint**					
All-cause mortality	0 (0)	0 (0)	14 (35)	5 (12.5)	<0.0001
**Secondary endpoints**					
Nervous system dysfunction	0 (0)	5 (12.5)	17 (42.5)	15 (37.5)	<0.0001
Delirium	0 (0)	5 (12.5)	16 (40.0)	13 (32.5)	<0.0001
Stroke	0 (0)	0 (0)	1 (2.5)	2 (5.0)	0.29
Acute heart failure	0 (0)	2 (5.0)	13 (32.5)	4 (10.0)	<0.0001
Acute lung injury	0 (0)	1 (2.5)	18 (45)	5 (12.5)	<0.0001
Acute kidney failure	0 (0)	0 (0)	4 (10.0)	1 (2.5)	0.031

Values are n (%), unless otherwise noted.
