# Supplementary material for: Specific immune landscape of heatstroke distinguished from sepsis and aseptic inflammation
Source: Int J Med Sci. 2025 Feb 26;22(6):1450–64. doi: 10.7150/ijms.108212 (PMC11898847; doi:10.7150/ijms.108212)
Supplement: Supplementary file 1 — Supplementary materials and datasets. [file ijmsv22p1450s1.zip › supplementary materials-final version.docx]

**Supplementary materials**

**For**

**Specific immune landscape of heatstroke distinguished from sepsis and aseptic inflammation**

Juan Wu ^1,#^, Zhenzhen Cheng ^2,#^, Sha Yang ^3^, Qinjuan Wu ^4^, Ping Yang ^5^, Xinyi Liao ^6^, Tao Cheng ^7^, Wenxia Huang ^8^, Yuan Zhu ^9^, Zongcheng Tang ^10^, Min Yan ^11^, Rong Yao ^12,^*, Lei Du ^13,^*

^1^ Department of Anesthesiology, West China Hospital, Sichuan University, Chengdu 610041, Sichuan, China. Email: 1436203224@qq.com

^2^ Department of Anesthesiology, West China Hospital, Sichuan University, Chengdu 610041, Sichuan, China and Department of Anesthesiology, Second Affiliated Hospital, Zhejiang University School of Medicine, Hangzhou 310000, Zhejiang, China. Email: chengzhenzhen@zju.edu.cn

^3^ Department of Emergency Medicine, West China Hospital, Sichuan University, Chengdu 610041, Sichuan, China. Email: 1414876149@qq.com

^4^ Department of Anesthesiology, Cheng Du Second People’s Hospital, Chengdu 610000, Sichuan, China. Email: [877035058@qq.com](mailto:877035058@qq.com)

^5^ Department of Anesthesiology, West China Hospital, Sichuan University, Chengdu 610041, Sichuan, China and Department of Anesthesiology, Chongqing University Three Gorges Hospital, Chongqing 404100, China. Email: sunflower_yp@outlook.com

^6^ Department of Anesthesiology, West China Hospital, Sichuan University, Chengdu 610041, Sichuan, China. Email: liaoxinyikk@163.com

^7^ Department of Emergency Medicine, West China Hospital, Sichuan University, Chengdu 610041, Sichuan, China. Email: chengtao@wchscu.edu.cn

^8^ Health Management Center, General Practice Medical Center, West China Hospital, Sichuan University, Chengdu 610041, Sichuan, China. Email: 1437454655@qq.com

^9^ Health Management Center, General Practice Medical Center, West China Hospital, Sichuan University, Chengdu 610041, Sichuan, China. Email: 359925690@qq.com

^10^ Department of Anesthesiology, West China Hospital, Sichuan University, Chengdu 610041, Sichuan, China. Email: [329595085@qq.com](mailto:329595085@qq.com)

^11^ Department of Anesthesiology, Second Affiliated Hospital, Zhejiang University School of Medicine, Hangzhou 310000, Zhejiang, China. Email: zryanmin@zju.edu.cn

^12^ Department of Emergency Medicine, West China Hospital, Sichuan University, Chengdu 610041, Sichuan, China. Email: [yaorong@wchscu.cn](mailto:yaorong@wchscu.cn)

^13^ Department of Anesthesiology, West China Hospital, Sichuan University, Chengdu 610041, Sichuan, China. Email: [dulei@scu.edu.cn](mailto:dulei@scu.edu.cn)

^#^ Juan Wu and Zhenzhen Cheng contributed equally to this study.

*** Corresponding authors:**

Lei Du MD PhD, Department of Anesthesiology, West China Hospital, Sichuan University, No. 37 Guo Xue Alley, Wuhou District, Chengdu 610041, Sichuan, China, E-mail: dulei@scu.edu.cn

Rong Yao MD PhD, Department of Emergency Medicine, West China Hospital, Sichuan University, No. 37 Guo Xue Alley, Wuhou District, Chengdu 610041, Sichuan, China, E-mail: [yaorong@wchscu.cn](mailto:yaorong@wchscu.cn).

1. **Materials and Methods**

**1.1 Study design and setting**

The protocol for this case-control exploratory study^[54]^ was approved by the Medical Ethics Committee of West China Hospital, Sichuan University (2022-1408) and registered at the Chinese Clinical Trial Registry (www.chictr.org.cn) under accession code ChiCTR-2200066952. All participants were recruited from West China Hospital of Sichuan University between January 1, 2023 and October 31, 2023. Written consent was obtained from all participants or their next of kin.

**1.2 Selection of Participants**

Participants with heatstroke or sepsis were prospectively recruited from the Department of Emergency Medicine, while patients undergoing cardiopulmonary bypass were recruited from the Department of Anesthesiology. Healthy controls were recruited from the Health Management Center. Inclusion and exclusion criteria are detailed in **Table S4**. Heatstroke was diagnosed as exertional heatstroke if the individual was otherwise healthy, relatively young and had been engaging in vigorous exercise in a hot or temperate environment when symptoms appeared. Alternatively, heatstroke was diagnosed as classic heatstroke if the individual was older and/or had pre-existing illness and the condition could be attributed to simple exposure to high ambient temperature, especially in the presence of high humidity ^[3]^.

The other three groups were matched with heatstroke patients in the ratio 1:1 based on age and sex using propensity score matching based on nearest-neighbor matching and a caliper width of 0.2 of the standard deviation of the logit of the propensity score ^[55]^. We did not estimate minimal sample size for this study because it was intended to be exploratory ^[54, 56]^.

**1.3 Endpoints**

The main purpose of this study was to identify heatstroke-specific immune system characteristics. During follow-up period, the primary endpoint was all-cause mortality within 30 days, and the secondary endpoints were the incidence of nervous system dysfunction^[57]^ (including stroke and delirium), acute heart failure^[58]^, acute kidney failure^[59]^ or acute lung injury^[60]^. Diagnostic criteria for these conditions are shown in **Table S5**.

**1.4 Data collection**

Demographic and clinical data at baseline were collected for all four groups. Scores on the Sequential Organ Failure Assessment^[61]^ and Glasgow Coma Scale^[62]^ were determined for patients with heatstroke or sepsis as described in **Table S6-7**. Lymphopenia was defined as absolute lymphocyte counts ≤ 1.1 × 10^9 cells/liter ^[49]^. Patients with heatstroke or sepsis were followed up for 30 days after admission, while cardiopulmonary bypass patients were followed up for 30 days after surgery as described ^[54]^.

**1.5 Laboratory measurements**

**1.5.1 Blood collection**

Venous blood (10 mL) was collected into EDTA-coated tubes within 24 h after screening in the case of patients with heatstroke or sepsis, at the end of cardiopulmonary bypass in the case of patients with aseptic inflammation, or during health check-ups in the case of healthy controls. The blood was analyzed using multi-channel spectral flow cytometry, protein arrays to detect cytokines in plasma, and sc-RNA seq (10x Genomics 5' platform).

**1.5.2 Multichannel spectral flow cytometry**

Venous blood (8 mL) was collected into tubes coated with EDTA and, within 4 h, treated with an in-house buffer [8.02 g NH4Cl, 0.84 g NaHCO3 and 0.37 g sodium EDTA per 1 L of phosphate-buffered saline (PBS); all reagents from Sigma-Aldrich (USA)]. The procedure was conducted as described ^[54]^. Briefly, aliquots of the resulting leukocyte suspension (100 μL) were labelled separately with antibodies against markers (**Table S8**) on the surface of immune cells for 30 min at room temperature in the dark, and one leukocyte suspension tube without antibodies for blank control. Then samples were centrifuged at 300 g at 4 °C for 5 min, the supernatant was discarded, the pellet was resuspended in 300 μL PBS, and the suspension was analyzed using FACSLyric ™ flow cytometer (BD Bioscience). We run quality control program in FACSLyric ™ flow cytometer before detecting samples, and samples were detected after passing it. Samples of staining single antibody were used for regulating compensation. Data were analyzed using FlowJo software (Treestar, USA), which was gated to exclude debris, dead cells and doublets while retaining single live cells. The technician performing flow cytometry was blinded to sample identity.

**1.5.3 Cytokine levels in plasma based on protein arrays**

We assayed levels of 40 cytokines in blood using the Quantibody Human Inflammation Array 3 kit (catalog no. OAH-INF-3, RayBioTech, USA). The procedure was performed based on the manufacturer’s instructions. Briefly, blood was centrifuged at 4000 rpm at 4 °C for 15 min, plasma was collected, and 100 μL plasma was incubated on the array overnight at 4 °C. The array was washed at room temperature five times with wash buffer I and twice with wash buffer II, incubated at room temperature with the biotinylated antibody cocktail for 2 h, again washed at room temperature five times with wash buffer I and two times with wash buffer II, and incubated with Cy3-conjugated streptavidin in the dark at room temperature for 1 h. The array was washed at room temperature five times with wash buffer I and two times with wash buffer II, then allowed to dry in the dark. Cy3 signal at 532 nm was measured using an InnoScan 300 system (Innopsys, France) at a spatial resolution of 10 µm. Signals were normalized to cell number. Antibodies were arranged on the array, in quadruplicate rows, as shown in **Table S9**.

**1.5.4 Single-cell RNA sequencing**

Venous blood (5 mL) from two individuals in each of the four groups was sampled into EDTA-coated tubes, and the eight samples were transferred to the clinical laboratory on ice, then subjected within 8 h after sampling to centrifugation through a Ficoll-Hypaque density gradient in order to isolate peripheral blood mononuclear cells. Samples in which the viability of mononuclear cells exceeded 80% were subjected to sc-RNA seq on a Chromium instrument using the Chromium Single-Cell 3′ kit (version 3; 10X Genomics, Pleasanton, CA, USA). Libraries from 8000 single cells per group were prepared according to the manufacturer's procedure and sequenced on an NovaSeq 6000 sequencing system (Illumina, San Diego, CA, USA) using paired-end 150-bp multiplexing at LC-Bio Technology (Hangzhou, China) to a depth of at least 20,000 reads per cell.

- *Quality control*

Sequences were analyzed by CellRanger 7.0.0 (10X Genomics) to extract information about gene expression information for each immune cell type of interest. Reads were aligned to a reference genome using the STAR aligner (github.com/alexdobin/STAR), which takes splicing into account. The resulting sequence alignments were loaded onto Seurat 4.1.1 software ([satijalab.org/seurat/](https://satijalab.org/seurat/)) for dimensionality reduction and clustering. Data were retained in the final analysis only for cells in which expression was detected for more than 500 genes, no more than 25% of which were mitochondrial. Multiplet cells were removed from sequencing data using DoubletFinder 2.0.3 ([github.com/chris-mcginnis-ucsf/DoubletFinder](https://github.com/chris-mcginnis-ucsf/DoubletFinder)). Expression levels of genes were visualized using the LogNormalize algorithm in the “Normalization” function of Seurat. Normalized expression levels were subjected to principal component analysis, and the 10 principal components that explained most of the observed variation were used to cluster samples and perform t-distributed stochastic neighbor embedding (t-SNE). Batch effects across samples were corrected using Harmony 0.1.0 ([www.nuget.org/packages/Harmony/0.1.0](http://www.nuget.org/packages/Harmony/0.1.0)).

- *Differential expression analysis*

Differential expression analysis for target immune cell types between different groups was performed using the bimod-test as implemented in the “FindMarkers” function of the Seurat V4.1.1 package. Differential expression genes were identified according to the following criteria: (1) a log_2_fold change ≥ 0.26, (2) p < 0.01, and (3) >1% of cells in either test group. Heatstroke-specific genes were defined as those that were differentially expressed in all pairwise comparisons of individuals with heatstroke against each of the three other groups of individuals.

- Enrichment of heatstroke-specific genes in Gene Ontology terms

Heatstroke-specific genes that were associated with GO terms (www.geneontology.org) at significantly greater frequency than the genome as a whole based on the hypergeometric test were considered to be enriched in those terms. The top 100 terms in which heatstroke-specific genes across various immune cell types showed the strongest enrichment for which p < 0.05 were ranked according to ascending p value.

- *GSEA*

GSEA was performed on single-cell RNA sequences using the “clusterProfiler” routine in R software (version 4.3.0, [www.r-project.org/](http://www.r-project.org/)) and was based on GO terms.

- *Cell-cell communication*

To investigate communication among T cells, B cells, monocytes and NK cells in the four groups of samples, we used CellPhoneDB (version 3; [www.cellphonedb.org](http://www.cellphonedb.org)) to identify instances when at least 10% of immune cells in one cluster, defined based on sc-RNA seq, expressed the ligand of a receptor that was expressed by at least 10% of immune cells in another cluster. Pairwise comparisons between cell subsets were performed by randomly permuting cluster labels for all cells 1000 times automatically, and the p value for each receptor-ligand in every cluster–cluster interaction was computed using a null distribution. The strength of receptor-ligand interactions was defined as the 'mean value', calculated by averaging the mean expressions of the genes encoding each ligand and its corresponding receptor.

**1.6 Statistical analysis**

Data were analyzed statistically using SPSS 25 (IBM, Armonk, NY, USA), and figures were prepared using Graphpad Prism 9.4.1 (Boston, MA, USA). Continuous data were tested for normality using Kolmogorov-Smirnov test and presented as mean ± SD if normally distributed or as median (interquartile range) otherwise. Differences among four groups were assessed for significance using the Kruskal-Wallis test or one-way ANOVA, among two groups were using the Mann-Whitney test or independent *t* test. Categorical data were reported as number (%), and intergroup differences were assessed using Fisher’s exact test or the chi-squared test. The ability of relative abundance of immune cell types or subpopulations to distinguish heatstroke from sepsis was assessed in terms of the area under receiver operating characteristic curves. Unless otherwise noted, results associated with p < 0.05 were considered significant.

1. **Supplementary figures and tables**


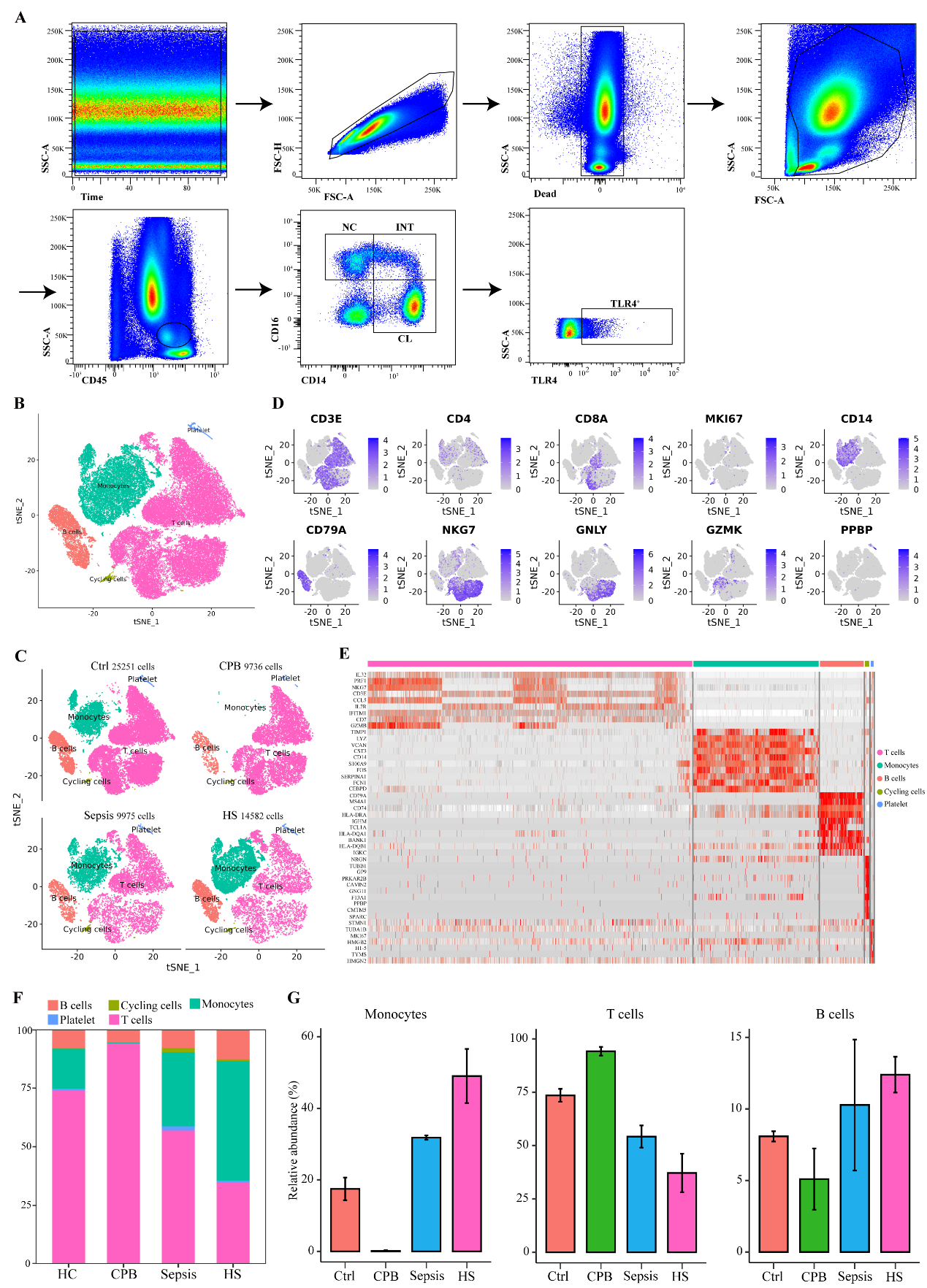


**Figure S1.** Five types of immune cells in the blood of healthy controls (Ctrl), patients undergoing cardiopulmonary bypass (CPB), and patients with heatstroke (HS) or sepsis (Sepsis). Clusters were identified from single-cell RNA sequencing.

**(A)** Gating strategy to analyze monocytes using flow cytometry. CL, classical monocytes; INT, intermediate monocytes; NC, nonclassical monocytes.

**(B-C)** Five types of immune cells were presented as (B) a whole and (C) in groups based on t-distributed stochastic neighbor embedding (t-SNE). A total of 25251 cells in the Ctrl group, 9736 cells in the CPB group, 9975 cells in the Sepsis group, and 14582 cells in the HS group passed the quality control threshold and were assigned to five types of immune cells. Each dot in t-SNE represents a cell, which is colored according to cell type.

**(D)** Clusters based on expression of canonical marker genes.

**(E)** Expression heatmap of canonical marker genes across the five types of immune cells.

**(F-G)** Relative abundances of the different types of immune cells (n=2 in each group). Median with IQR is shown. Significant differences between groups were assessed using Kruskal- Wallis test.


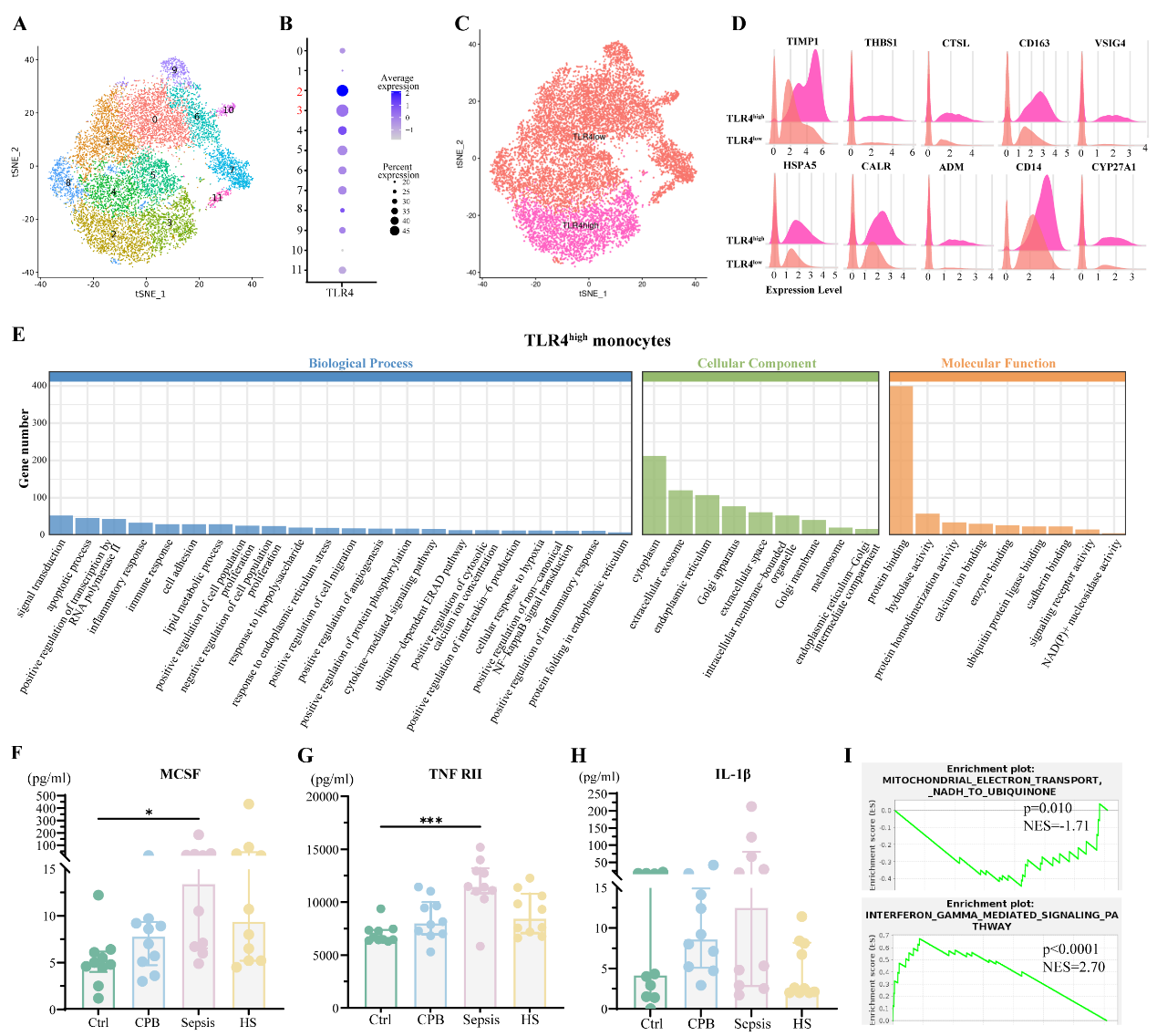


**Figure S2.** The signature of monocytes expressing high TLR4 in heatstroke. Samples were those as defined in Figure S1.

1. Twelve clusters in monocytes identified by t-distributed stochastic neighbor embedding (t-SNE).

Each dot represents a cell, which is colored according to subpopulation.

1. The dot plot of TLR4 expression among 12 clusters. Clusters 2 and 3 exhibited the highest

expression of TLR4 and were identified as TLR4^high^ monocytes, while the remaining clusters were categorized as TLR4^low^ monocytes.

1. The t-SNE projection of TLR4^high^ and TLR4^low^ monocytes.
2. Ridge map depicting the top 10 most significantly upregulated genes identified by

"findallmarker" analysis in TLR4^high^ monocytes versus TLR4^low^ monocytes.

1. A selection of Gene Ontology terms enriched in differentially expressed genes in TLR4^high^

monocytes compared with TLR4^low^ monocytes.

**(F-H)** Levels of the pro-inflammatory cytokines (F) macrophage colony-stimulating factor (MSCF), (G) tumor necrosis factor receptor II (TNF-II) and (H) IL-1β in plasma (n=10 in each group). Median with IQR is shown. Significant differences between groups were assessed using Kruskal- Wallis test, *p ≤ 0.05, and ***p < 0.001.

1. Gene set enrichment analysis of heatstroke-specific genes differentially expressed in TLR4^high^

monocytes.

CPB, cardiopulmonary bypass; HS, heatstroke; TLR4, toll-like receptor 4.


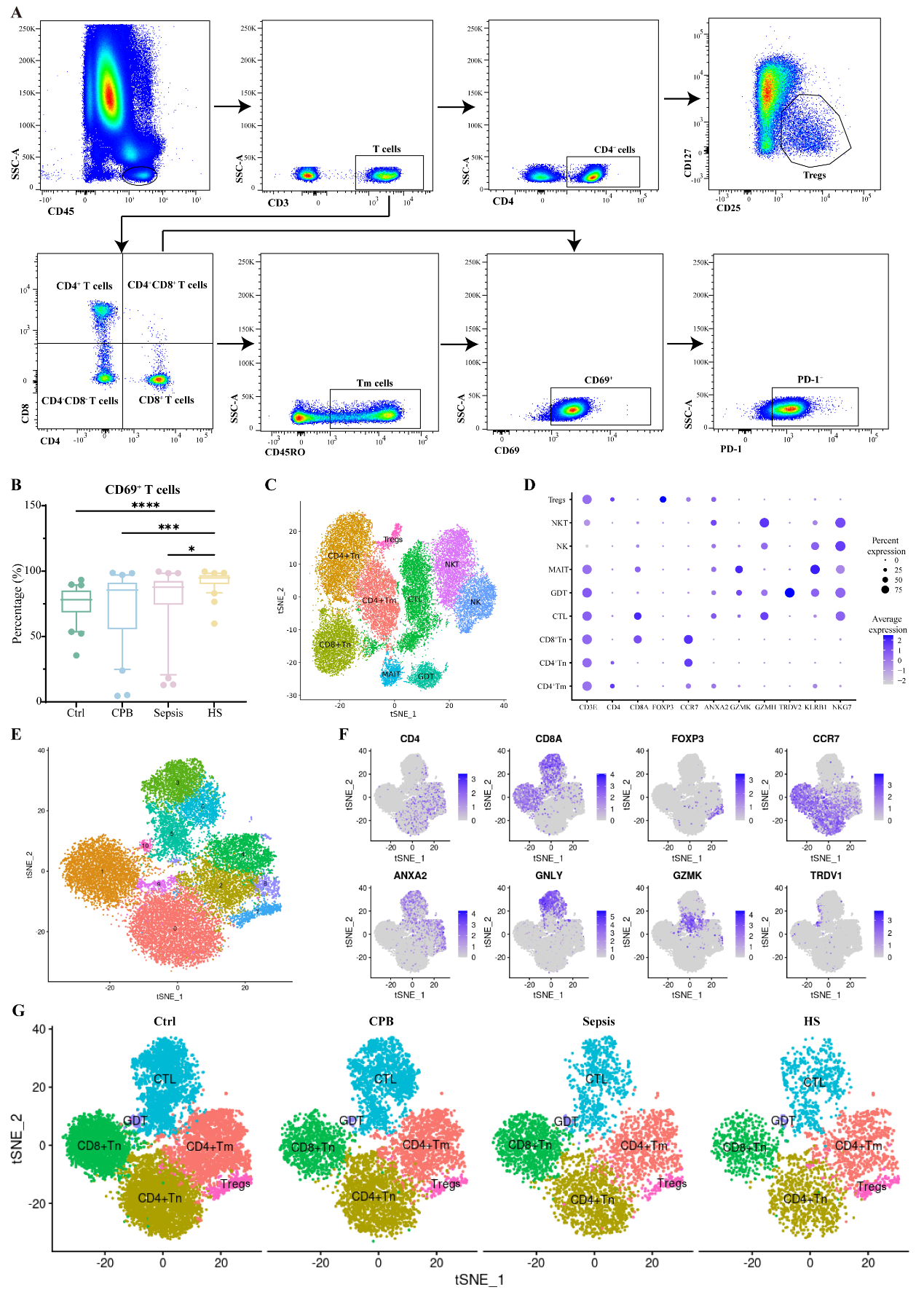


**Figure S3.** Analysis of subpopulations of CD4^+^ and CD8^+^ T cells based on single-cell RNA sequencing. Samples were those as defined in Figure S1.

**(A)** Gating strategy for flow cytometry.

**(B)** The box plots of relative abundance of CD69-expressing cells among T cells (n=40 in each group). Significant differences between groups were assessed using Kruskal- Wallis test, *p ≤ 0.05, ***p < 0.001 and ****p < 0.0001.

**(C)** Nine subpopulations of T cells based on t-distributed stochastic neighbor embedding (t-SNE). Each dot represents a cell, which is colored according to cell type. Tregs, regulatory T cells; Tm, memory T cells; NK, natural killer cells; CTL, cytotoxic T lymphocytes; GDT, γδ T cells; MAIT, mucosal-associated invariant T cells.

**(D)** Dot plot of expression of canonical marker genes across the nine subpopulations.

**(E-F)** Eleven clusters of CD4^+^ and CD8^+^ T cell based on t-SNE, with projections according to expression of canonical marker genes.

(**G)** Subpopulations of CD4^+^ and CD8^+^ T cells were presented in groups based on t-SNE.


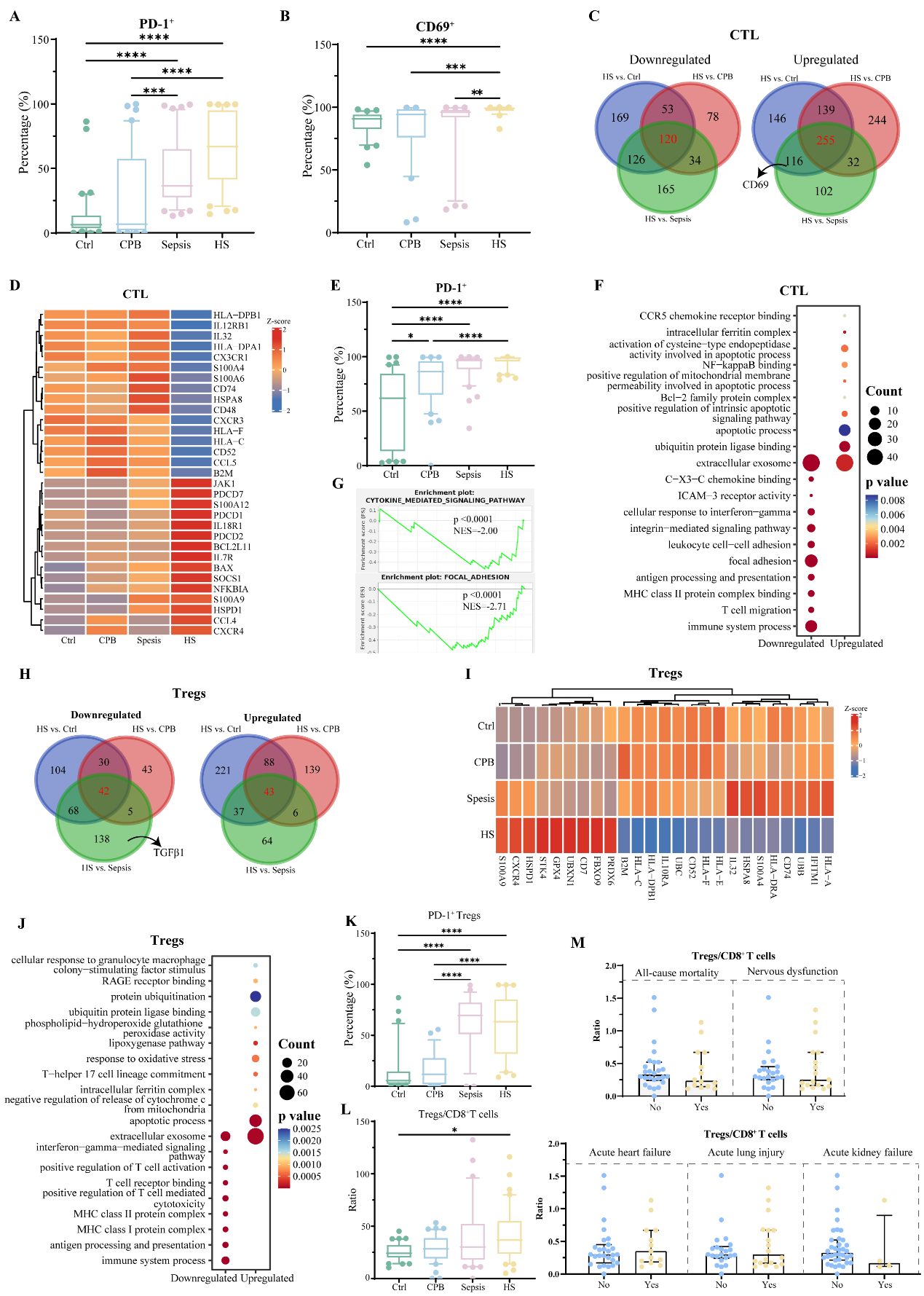


**Figure S4.** Heatstroke is associated with T cell exhaustion. Samples were those as defined in Figure S1.

**(A-B)** Box plots of relative abundance of (A) PD-1^+^ and (B) CD69^+^ among CD4^+^ Tm cells (n=40 in each group). Significant differences between groups were assessed using Kruskal- Wallis test, **p < 0.01, ***p < 0.001 and ****p < 0.0001.

1. Overlap of differentially expressed genes in cytotoxic T lymphocytes (CTL) in three

pairwise comparisons. Red numbers refer to heatstroke-specific genes.

1. Expression heatmap of heatstroke-specific genes differentially expressed in CTL.
2. The box plots of relative abundance of the PD-1^+^ among CD69^+^CD8^+^ T cells (n=40 in each group).

**(F-G)** Heatstroke-specific genes differentially expressed in CTL were analyzed for (F) enrichment in Gene Ontology terms and (G) gene set enrichment analysis.

1. Overlap of differentially expressed genes in regulatory T cells (Tregs) in three pairwise

comparisons. Red numbers refer to heatstroke-specific genes.

**(I-J)** Heatstroke-specific genes differentially expressed in Tregs were analyzed for (I) expression and (J) enrichment in Gene Ontology terms.

1. Box plots of relative abundance of the PD-1^+^ among Tregs.
2. Box plots of ratios of the abundance of Tregs to abundance of CD8^+^ T cells.
3. Ratios of the abundance of Tregs to abundance of CD8^+^ T cells in sepsis patients stratified by

whether they died or suffered nervous system dysfunction, acute heart failure, or acute lung injury within 30 days after admission. Median with IQR is shown. Significant differences between groups were assessed using Mann-Whitney test.

CPB, cardiopulmonary bypass; HS, heatstroke; PD-1, programmed cell death protein-1; Tm, memory T cells.


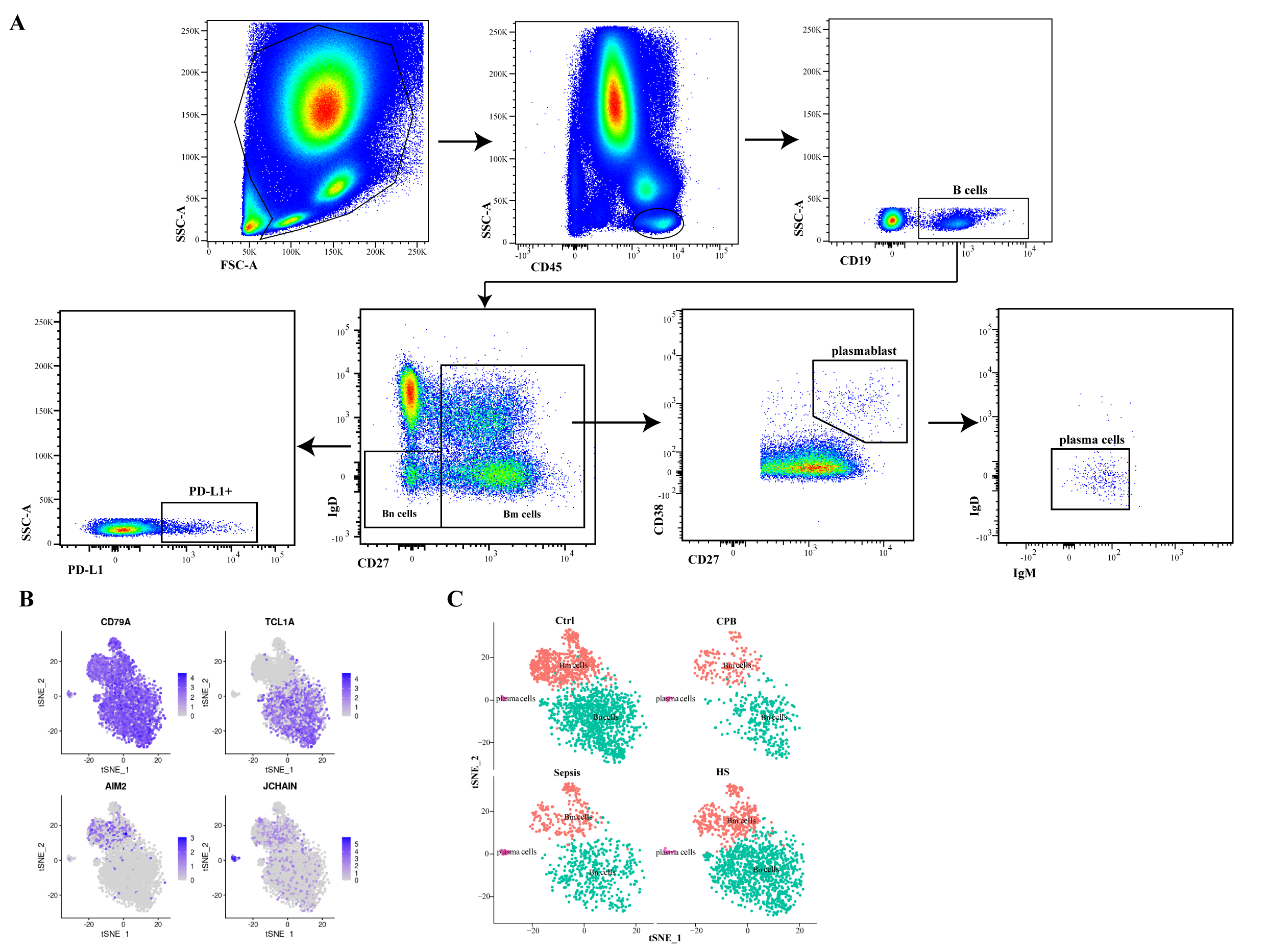


**Figure S5**. B cell signature in heatstroke. Samples were those as defined in Figure S1.

1. Gating strategy for flow cytometry.
2. Expression of canonical markers based on t-distributed stochastic neighbor embedding (t-SNE).
3. Subpopulations of B cells were presented in groups based on t-SNE. Each dot represents a cell,

which is colored according to cell type.

Bn, naïve B cells; Bm, memory B cells; CPB, cardiopulmonary bypass; HS, heatstroke.


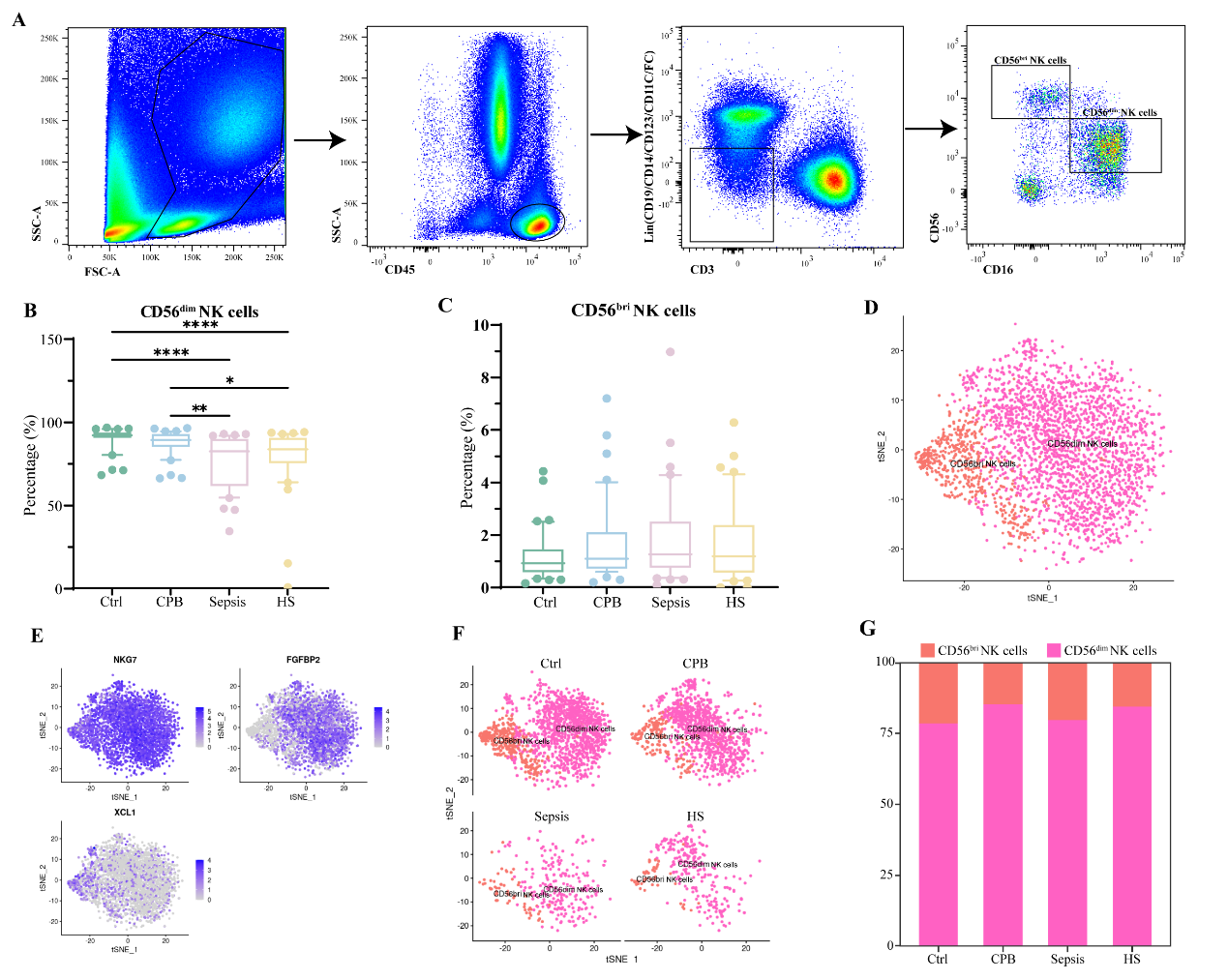


**Figure S6.** NK cell signature in heatstroke. Samples were those as defined in Figure S1.

1. Gating strategy for flow cytometry.

**(B-C)** Box plots of relative abundance of the natural killer (NK) cell subpopulations (B) CD56^dim^ and (C) CD56^bri^ among NK cells (n=40 in each group). Significant differences between groups were assessed using Kruskal- Wallis test, *p ≤ 0.05, **p < 0.01, and ****p < 0.0001.

**(D-E)** NK cell subpopulations based on t-distributed stochastic neighbor embedding (t-SNE), with projections according to expression of canonical marker genes. Each dot represents a cell, which is colored according to cell type.

1. Subpopulations of NK cells were presented in groups based on t-SNE. Each dot represents a

cell, which is colored according to cell type.

1. Relative abundance of CD56^dim^ and CD56^bri^ NK cells, based on single-cell RNA sequencing.

bri, bright; CPB, cardiopulmonary bypass; HS, heatstroke.


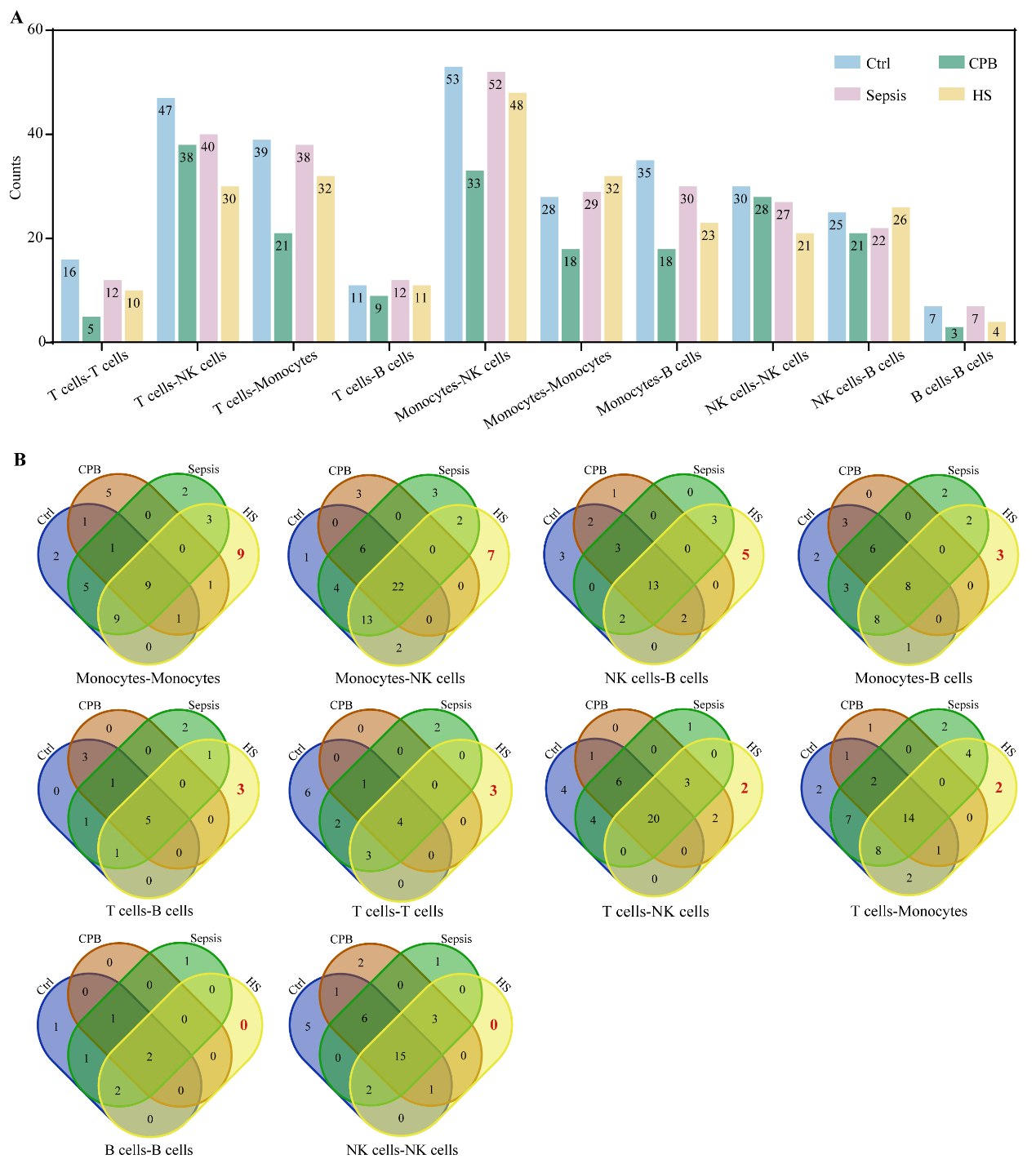


**Figure S7**. Specific cell-cell interaction in heatstroke. Samples were those as defined in Figure S1.

1. The counts of receptor-ligands between immune cells types among four groups.
2. Overlap of receptor-ligands pairs in four groups. Red numbers indicate the numbers of receptor

ligands interactions that appeared only in heatstroke.

CPB, cardiopulmonary bypass; HS, heatstroke; NK cells, natural killer cells.

| **Table S1.** Comparison of clinicodemographic characteristics between patients with exertional or classic heatstroke | | | |
| --- | --- | --- | --- |
| **Characteristic** | **Exertional**  **(n=22)** | **Classic**  **(n=18)** | **p** |
| **Basic information** |  |  |  |
| Age, yr | 45 (32-59) | 69 (59-74) | <0.0001 |
| Sex |  |  | 0.001 |
| Male | 19 (86) | 6 (33) |  |
| Female | 3 (14) | 12 (67) |  |
| Body mass index, kg/m^2^ | 24.5 (22.8-26.3) | 24.9 (21.2-26.5) | 0.98 |
| Duration from symptom onset until admission, h | 24 (14-72) | 24 (4-60) | 0.44 |
| SOFA score | 8 (5-13) | 9 (6-14) | 0.73 |
| Glasgow Coma Scale score | 6 (3-12) | 6 (3-12) | 0.79 |
| **Comorbidities** |  |  |  |
| Hypertension | 3 (14) | 6 (33) | 0.25 |
| Diabetes | 2 (9) | 2 (11) | 1.0 |
| **Laboratory results** |  |  |  |
| Red blood cells, 10^12^/L | 3.5 (2.8-4.0) | 3.7 (2.9-4.1) | 0.73 |
| Hemoglobin, g/L | 104 (81-125) | 112 (93-124) | 0.75 |
| Platelets, 10^9^/L | 92 (44-170) | 73 (44-107) | 0.45 |
| White blood cells, 10^9^/L | 8.7 (6.8-9.9) | 9.1 (7.3-12.7) | 0.70 |
| Monocytes, % | 7.2 (4.7-10.4) | 8.3 (5.6-10.8) | 0.51 |
| Neutrophils, % | 82.6 (75.9-88.2) | 81.3 (66.4-87.1) | 0.34 |
| Lymphocytes, % | 9.4 (5.1-13.8) | 9.4 (4.6-21.8) | 0.62 |
| Lymphocytes, 10^9^/L | 1.0 (0.4-1.2) | 0.8 (0.6-1.4) | 0.64 |
| **Flow cytometry results** |  |  |  |
| TLR4^+^ monocytes, % | 43.1 (16.9-57.8) | 35.0 (7.0-50.0) | 0.21 |
| Mean TLR4 fluorescence in monocytes | 117.0 (84.8-149.8) | 91.2 (62.6-128.0) | 0.12 |
| TLR4^+^ classical monocytes, % | 41.3 (10.0-52.8) | 28.0 (3.6-49.5) | 0.30 |
| Mean TLR4 fluorescence in classical monocytes | 114.5 (81.9-134.5) | 106.0 (65.2-128.0) | 0.33 |
| T cells, 10^9^/L | 0.6 (0.3-0.9) | 0.6 (0.2-0.9) | 0.93 |
| CD69^+^ T cells, % | 90.0 (58.6-96.6) | 93.3 (80.1-96.5) | 0.70 |
| CD4^+^ CD69^+^ T cells, % | 92.3 (57.9-97.9) | 94.9 (81.6-97.3) | 0.82 |
| CD8^+^ CD69^+^ T cells, % | 85.9 (45.5-94.8) | 89.6 (71.4-95.9) | 0.46 |
| B cells, 10^9^/L | 0.1 (0.0-0.2) | 0.1 (0.0-0.2) | 0.84 |
| PD-L1^+^ B cells, % | 8.4 (0.4-19.4) | 9.7 (1.0-13.0) | 0.82 |
| PD-L1^+^ Bn cells, % | 21.0 (8.0-31.1) | 22.8 (4.5-36.7) | 0.64 |
| PD-L1^+^ Bm cells, % | 4.3 (0.2-6.6) | 4.1 (1.0-8.1) | 0.99 |
| NK cells, 10^9^/L | 0.0 (0.0-0.1) | 0.1 (0.0-0.2) | 0.24 |
| CD335^+^ NK cells, % | 23.4 (13.3-60.8) | 18.5 (11.5-31.8) | 0.27 |
| CD56^dim^ NK cells (%) | 82.2 (73.9-90.8) | 84.5 (75.1-91.3) | 0.72 |
| CD335^+^CD56^dim^ NK cells, % | 24.5 (12.6-59.8) | 18.9 (9.6-32.7) | 0.37 |
| CD335^+^CD56^bri^ NK cells, % | 64.5 (31.0-80.9) | 75.7 (34.7-94.4) | 0.37 |
| **Primary outcome** |  |  |  |
| All-cause mortality | 2 (9) | 3 (17) | 0.64 |
| **Secondary outcomes, n (%)** |  |  |  |
| Nervous system dysfunction | 9 (41) | 6 (33) | 0.75 |
| Delirium | 9 (41) | 4 (22) | 0.31 |
| Stroke | 0 (0) | 2 (11) | 0.20 |
| Acute heart failure | 1 (5) | 3 (17) | 0.31 |
| Acute lung injury | 3 (14) | 2 (11) | 1.00 |
| Acute kidney failure | 0 (0) | 1 (6) | 0.45 |

Values are n (%) or median (interquartile range), unless otherwise noted.

Bm, memory B; Bn, naïve B; bri, bright; NK, natural killer; PD-1, programmed death,1; PD-L1, programmed death-ligand 1; SOFA, sequential organ failure assessment; TLR4, toll-like receptor 4; Tm, memory T; Treg, regulatory T.

| **Table S2.** Median values and interquartile ranges for selected analytical results in the four groups | | | | |
| --- | --- | --- | --- | --- |
| **Result** | **Healthy controls** | **Cardiopulmonary bypass** | **Sepsis** | **Heatstroke** |
| White blood cells, 10^9^/L | 5.4 (4.5-7.0) | 9.8 (6.2-12.9) | 10.2 (7.4-15.5) | 8.8 (7.1-11.5) |
| Monocytes, % | 7.4 (6.2-9.0) | 2.2 (1.6-2.9) | 4.9 (2.7-6.9) | 7.5 (4.8-10.5) |
| TLR4^+^ monocytes, % | 7.0 (2.4-12.5) | 10.1 (4.8-19.1) | 14.4 (3.0-27.2) | 36.9 (14.8-54.5) |
| Mean TLR4 fluorescence in monocytes | 42.4 (32.4-56.5) | 47.5 (37.2-60.1) | 64.9 (49.5-80.6) | 107 (72.2-137.8) |
| TLR4^+^ classical monocytes, % | 1.4 (0.4-7.5) | 2.6 (1.2-6.1) | 5.1 (1.2-13.2) | 32.8 (8.8-51.6) |
| Mean TLR4 fluorescence in classical monocytes | 47.5 (35.9-60.4) | 49.5 (38.8-57.8) | 62.9 (50.4-83.5) | 109.0 (70.4-130.8) |
| Lymphocytes, 10^9^/L | 1.68 (1.22-1.99) | 1.80 (1.19-2.72) | 0.68 (0.36-1.04) | 0.91 (0.51-1.24) |
| T cells, 10^9^/L | 1.12 (0.7-1.29) | 0.99 (0.62-1.46) | 0.40 (0.22-0.70) | 0.58 (0.28-0.87) |
| CD69^+^ T cells, % | 78.1 (68.2-85.6) | 85.6 (55.3-91.5) | 87.8 (74.1-92.9) | 94.9 (89.9-97.4) |
| CD4^+^ CD69^+^ T cells, % | 83.5 (71.1-88.6) | 90.3 (59.8-95.3) | 90.0 (75.4-93.7) | 96.5 (91.8-98.5) |
| CD8^+^ CD69^+^ T cells, % | 69.7 (59.7-79.9) | 75.2 (43.8-90.4) | 83.7 (71.6-91.3) | 92.1 (86.8-95.9) |
| PD-1^+^ CD4^+^ Tm cells, % | 6.4 (3.4-13.8) | 6.8 (1.8-57.8) | 36.4 (27.4-65.4) | 66.9 (41.2-95.3) |
| CD69^+^ CD4^+^ Tm cells, % | 89.6 (80.7-94.9) | 91.1 (75.4-98.6) | 94.0 (66.4-97.4) | 98.3 (82.6-99.4) |
| PD-1^+^ CD8^+^ CD69^+^ T cells, % | 62.0 (13.1-84.5) | 86.4 (65.0-96.9) | 96.6 (88.3-98.5) | 98.6 (95.4-99.6) |
| PD-1^+^ Treg cells, % | 4.5 (1.9-16.8) | 10.1 (1.7-30.4) | 68.9 (41.1-81.1) | 64.1 (33.6-79.3) |
| Ratio of Treg cells to CD8^+^ cells | 24.2 (19.9-32.1) | 28.9 (22.1-40.2) | 36.7 (23.4-32.1) | 30.2 (19.9-52.4) |
| B cells, 10^9^/L | 0.10 (0.08-0.16) | 0.06 (0.03-0.09) | 0.06 (0.02-0.12) | 0.10 (0.04-0.17) |
| Bn cells, % | 6.8 (4.5-8.1) | 10.6 (7.9-15.3) | 10.8 (6.2-16.0) | 8.8 (5.8-14.6) |
| Bm cells, % | 35.2 (26.3-40.6) | 24.7 (17.7-32.0) | 45.0 (25.2-58.7) | 36.7 (25.9-50.0) |
| Plasma cells, % | 1.1 (0.5-2.0) | 0.9 (0.2-2.0) | 2.0 (0.6-4.5) | 0.9 (0.3-2.6) |
| PD-L1^+^ B cells, % | 1.6 (0.8-2.7) | 5.3 (2.3-10.4) | 5.2 (2.1-8.8) | 8.9 (0.6-15.6) |
| PD-L1^+^ Bn cells, % | 9.5 (5.5-13.9) | 22.9 (12.9-41.7) | 16.7 (9.6-30.7) | 21.0 (6.4-34.8) |
| PD-L1^+^ Bm cells, % | 0.8 (0.4-1.8) | 3.4 (1.5-7.3) | 2.9 (0.8-5.3) | 4.1 (0.5-7.1) |
| NK cells, 10^9^/L | 0.22 (0.13-0.36) | 0.27 (0.18-0.59) | 0.06 (0.03-0.13) | 0.04 (0.02-0.10) |
| CD56^dim^ NK cells, % | 92.2 (90.4-94.2) | 89.4 (85.0-93.2) | 82.5 (61.4-90.3) | 83.8 (75.2-91.0) |
| CD56^bri^ NK cells, % | 0.9 (0.6-1.5) | 1.1 (0.7-2.2) | 1.3 (0.7-2.5) | 1.2 (0.6-2.4) |
| CD335^+^ NK cells, % | 25.8 (14.9-44.2) | 63.4 (39.0-72.3) | 20.8 (12.3-45.8) | 20.1 (12.9-52.1) |
| CD335^+^CD56^dim^ NK cells, % | 24.2 (13.9-42.9) | 63.4 (38.3-72.3) | 20.6 (12.2-51.9) | 21.6 (11.7-54.9) |
| CD335^+^CD56^bri^ NK cells, % | 94.1 (86.9-97.3) | 94.3 (77.2-97.5) | 50.0 (27.6-83.5) | 68.8 (33.8-86.2) |

Bm, memory B; Bn, naïve B; bri, bright; NK, natural killer; Tm, memory T; Treg, regulatory T.

| **Table S3.** Canonical marker genes whose expression was used to define immune cell types and their subpopulations | | | | | |
| --- | --- | --- | --- | --- | --- |
| **Cell type** | **Marker** | | **Subpopulation** | **Marker** | |
|  | **Positive** | **Negative** |  | **Positive** | **Negative** |
| T cell | CD3E | / | CD4 Tn cells | CD4, CCR7 | ANXA2 |
|  |  |  | CD4 Tm cells | CD4, CCR7, ANXA2 |  |
|  |  |  | CD4 Treg | CD4, FOXP3 |  |
|  |  |  | CD8 Tn cells | CD8A, CCR7 |  |
|  |  |  | CTL | CD8A, GZMK/GNLY |  |
|  |  |  | NKT | NKG7 |  |
|  |  |  | GDT | TRDV2 |  |
|  |  |  | MAIT | KLRB1 |  |
| NK cells | NKG7 | CD3E | CD56^dim^ NK cells | FGFBP2 |  |
|  |  |  | CD56^bri^ NK cells | XCL1 |  |
| B cell | CD79A | / | Bn cells | TCL1A |  |
|  |  |  | Bm cells | AIM2 |  |
|  |  |  | Plasma cells | JCHAIN |  |
| Monocytes | CD14 | / | / |  |  |
| Platelets | PPBP | / | / |  |  |
| Cycling T cells | MKI67 | / | / |  |  |

Bm, memory B; Bn, naïve B; bri, bright; CTL, cytotoxic T lymphocytes; GDT, γδ T cells; MAIT, mucosal-associated invariant T cells; NK, natural killer; Tm, memory T; Tn, naïve T; Treg, regulatory T.

| Table S4. Inclusion and exclusion criteria for recruitment of subjects into this study. | | |
| --- | --- | --- |
| Group | **Inclusion** **criteria** | **Exclusion criteria** |
| Heatstroke | (1) Age ≥ 18 years  (2) History of exposure to extreme environmental heat or vigorous muscle exertion  (3) Core body temperature >40 ºC or symptoms of central nervous system (CNS) dysfunction, such as coma, delirium or convulsions  (4) Written informed consent to participate | (1) Pregnant women  (2) Comorbid with immune system disease within the six months prior to admission, including but not limited to acquired immune deficiency syndrome (AIDS), multiple myeloma, rheumatoid arthritis, systemic lupus erythematosus, multiple sclerosis, myasthenia gravis, scleroderma, Crohn's disease and ulcerative colitis;  (3) Comorbid with malignant tumor and hematological diseases  (4) History of organ or bone marrow transplantation  (5) History of immunosuppressive therapy, including glucocorticoids, soluble microbial products, polyclonal and monoclonal antibodies, antimetabolic or alkylating agents, or radiotherapy within the six months prior to admission. |
| Sepsis | (1) Age ≥ 18 years  (2) Suspected or known infection  (3) Sequential organ failure assessment (SOFA) score ≥ 2  (4) Written informed consent to participate |  |
| Cardiopulmonary bypass | (1) Age ≥ 18 years  (2) Valve replacement or coronary artery bypass under cardiopulmonary bypass  (3) Written informed consent to participate |  |
| Healthy controls | (1) Age ≥ 18 years  (2) Body mass index 18-24 kg/m^2^  (3) Health examination within the previous six months indicated no abnormality  (4) Written informed consent to participate |  |

| **Table S5.** Definitions of non-mortality outcomes | |
| --- | --- |
| **Outcome** | **Definition** |
| Nervous system dysfunction | Included stroke and delirium. Stroke was diagnosed based on symptoms of unconsciousness or focal neurologic deficit and the results of computerized tomography and magnetic resonance imaging. Delirium was diagnosed using the Confusion Assessment Method for the ICU. |
| Acute kidney failure | Stage 3 acute kidney injury according to KDIGO criteria, which define stage 3 as creatinine level in serum ≥ 4.0 mg/dL (353.60 µmol/L) or ≥ 3 times baseline within 7 days, or requirement for renal replacement therapy, or urine output <0.3 mL/kg/h for ≥ 24 h or anuria for ≥ 12 h. |
| Acute heart failure | Diagnosed according to symptoms of breathlessness, tiredness and fatigue; clinical signs; electrocardiographic abnormalities such as atrial fibrillation, Q waves, left ventricle hypertrophy, and a widened QRS complex; left ventricular ejection fraction (LVEF)≤40% based on echocardiography; and elevated levels of B-type natriuretic peptide (BNP) or NT-proBNP. |
| Acute lung injury | Defined as pH < 7.35 and PaCO_2_ > 45 mmHg or as PaO_2_/FiO_2_ < 200 based on invasive or non-invasive ventilation or arterial blood gas analysis. |

| **Table S6.** Scoring on the Sequential Organ Failure Assessment scale | | | | | |
| --- | --- | --- | --- | --- | --- |
| **Clinical variable** | **0** | **1** | **2** | **3** | **4** |
| PaO_2_/FiO_2_(mmHg) | > 400 | ≤ 400 | ≤ 300 | ≤ 200 | ≤ 100 |
|  |  |  |  | with respiratory support | |
| Platelets, 10^9^/L | > 150 | ≤ 150 | ≤ 100 | ≤ 50 | ≤ 20 |
| Bilirubin, μmol/L | < 20 | 20–32 | 33–101 | 102–204 | > 204 |
| Hypotension | MAP >70 mmHg | MAP < 70 mmHg | Dopamine ≤ 5 or dobutamine (any dose) ^a^ | Dopamine > 5 or epinephrine ≤ 0.1 or norepinephrine ≤ 0.1^a^ | Dopamine > 15 or epinephrine > 0.1 or norepinephrine > 0.1^a^ |
| Glasgow Coma Scale score ^c^ | 15 | 13-14 | 10-12 | 6-9 | <6 |
| Creatinine, μmol/L | < 110 | 110–170 | 171–299 | 300–440 | > 440 |
| Urinary output | - | - | - | < 500 ml/d | < 200 ml/d |

FiO_2_, fraction of inspired oxygen; MAP, mean arterial pressure; PaO_2_, partial pressure of oxygen

^a^ Catecholamine doses are µg/kg/min for at least 1 hour.

^c^ Glasgow Coma Scale scores range from 3-15; higher score indicates better neurological function.

| **Table S7.** Scoring on the Glasgow coma scale | | |
| --- | --- | --- |
| **Eye opening** | **Verbal response** | **Motor response** |
| 4. Spontaneous | 5. Oriented | 6. Obeys commands |
| 3. In response to speech | 4. Sentences | 5. Localizes pain |
| 2. In response to pain | 3. Words | 4. Flexion/withdrawal in response to pain |
| 1. No response | 2. Sounds | 3. Abnormal flexion in response to pain |
|  | 1. No response | 2. Extension in response to pain |
|  |  | 1. No response |
| The scores in each of the three columns are summed to give a total score. Total scores of 13-15 indicate mild head injury; 9-12, moderate; and 3-8, severe. | | |

| **Table S8. Staining panels for flow cytometry** | | |
| --- | --- | --- |
| **Fluorophore** | **Antigen** | **Catalog no.** |
| **T cell panel 1** | | |
| BV421 | BV421 Mouse Anti-Human CD279 (PD-1) (MIH4) | 564323 |
| BV510 | BV510 Mouse Anti-Human CD25 (M-A251) | 563352 |
| BV605 | BV605 Mouse Anti-Human CD45 (HI30) | 564047 |
| FITC | FITC Mouse anti-Human CD197 (CCR7) (150503) | 561271 |
| PE | PE Mouse Anti-Human CD127 (HIL-7R-M21) | 557938 |
| PerCP-Cy5.5 | PerCP-Cy5.5 Mouse Anti-HumanCD3 (UCHT1) | 560835 |
| PE-Cy7 | PE-Cy7 Mouse Anti-Human CD28 (CD28.2) | 560684 |
| APC | APC Mouse Anti-Human CD45RA (HI100) | 550855 |
| APC-Cy7 | APC-Cy7 Mouse Anti-Human CD4 (RPA-T4) | 557871 |
| APC-R700 | Fixable Viability Stain 700 | 564997 |
| **T cell panel 2** | | |
| BV421 | BV421 Mouse Anti-Human CD279 (PD-1) (MIH4) | 564323 |
| BV510 | BV510 Mouse Anti-Human CD8 (SK1) | 563919 |
| BV605 | BV605 Mouse Anti-Human CD45 (HI30) | 564047 |
| FITC | FITC Mouse Anti-Human CD38 (HIT2) | 555459 |
| PE | PE Mouse Anti-Human CD69 (FN50) | 557050 |
| PerCP-Cy5.5 | PerCP-Cy5.5 Mouse Anti-Human CD3 (UCHT1) | 560835 |
| PE-Cy7 | PE-Cy7 Mouse Anti-Human CD28 (CD28.2) | 560684 |
| APC | APC Mouse Anti-Human CD45RO (UCHL1) | 559865 |
| APC-Cy7 | APC-Cy7 Mouse Anti-Human CD4 (RPA-T4) | 557871 |
| APC-R700 | Fixable Viability Stain 700 | 564997 |
| **B cells** | | |
| BV421 | BV421 Mouse Anti-Human CD27 (M-T271) | 562513 |
| BV510 | BV510 Mouse Anti-Human CD19 (SJ25C1) | 562947 |
| BV605 | BV605 Mouse Anti-Human CD45 (HI30) | 564047 |
| FITC | FITC Mouse Anti-Human CD38 (HIT2) | 555459 |
| PE | PE Mouse Anti-Human IgG (G18-145) | 555787 |
| PerCP-Cy5.5 | PerCP-Cy5.5 Mouse Anti-HumanIgM (G20-127) | 561285 |
| PE-Cy7 | PE-Cy7 Mouse Anti-Human CD274 (MIH1) | 558017 |
| APC | APC anti-human CD80 | 305220 |
| APC-Cy7 | APC-H7 Mouse Anti-Human IgD (IA6-2) | 561305 |
| APC-R700 | Fixable Viability Stain 700 | 564997 |
| **Monocytes** | | |
| BV421 | BV421 Mouse Anti-Human TLR4 (CD284) (TF901) | 564401 |
| BV510 | BV510 Mouse Anti-Human CD40 (5C3) | 563456 |
| BV605 | BV605 Mouse Anti-Human CD45 (HI30) | 564047 |
| FITC | FITC Mouse Anti-Human CD14 (M5E2) | 555397 |
| PE | PE Mouse Anti-Human HLA-DR (TU36) | 555561 |
| PerCP-Cy5.5 | PerCP-Cy5.5 Mouse Anti-Human CD80 (B7-1) (2D10.4) | 567437 |
| PE-Cy7 | PE-Cy7 Mouse Anti-Human CD274 (MIH1) | 558017 |
| APC | Alexa Fluor 647 Mouse Anti-Human CD163 (GHI/61) | 562669 |
| APC-Cy7 | APC-H7 Mouse Anti-Human CD16 (3G8) | 560195 |
| APC-R700 | Fixable Viability Stain 700 | 564997 |
| **NK cells** | | |
| BV421 | BV421 Mouse Anti-Human CD57(NK-1) | 563896 |
| BV510 | BV510 Mouse Anti-Human CD335 (NKp46) (9E2/NKp46) | 564064 |
| BV605 | BV605 Mouse Anti-Human CD45 (HI30) | 564047 |
| FITC | FITC Mouse Anti-Human CD11c(B-ly6), FITC Mouse anti-Human CD123(7G3), FITC Mouse Anti-Human CD19(HIB19), FITC Mouse Anti-Human CD14(M5E2), Human BD Fc Block (Fc1.3216) | 561355,558663,555412,555397,564219 |
| PE | PE Mouse Anti-Human CD127 (HIL-7R-M21) | 557938 |
| PerCP-Cy5.5 | PerCP-Cy5.5 Mouse Anti-HumanCD3 (UCHT1) | 560835 |
| PE-Cy7 | PE-Cy7 Mouse Anti-Human CD56 (NCAM-1) (B159) | 557747 |
| APC | APC Mouse Anti-Human CD314 (NKG2D) (1D11) | 558071 |
| APC-Cy7 | APC-H7 Mouse Anti-Human CD16 (3G8) | 560195 |
| APC-R700 | Fixable Viability Stain 700 | 564997 |

* The source of all antibodies was BD Pharmingen (Franklin, NJ, USA), except APC-conjugated antibody against human CD80, which was purchased from Biolegend (San Diego, CA, USA).

| **Table S9.** Organization of the 40 antibodies on the protein microarray used to assay plasma from subjects. | | | | | | | | | | | | |
| --- | --- | --- | --- | --- | --- | --- | --- | --- | --- | --- | --- | --- |
|  | 1 | 2 | 3 | 4 | 5 | 6 | 7 | 8 | 9 | 10 | 11 | 12 |
| A | POS1 | | | | POS2 | | | | CXCL13 | | | |
| B | CCL11 | | | | CCL24 | | | | GCSF | | | |
| C | GM-CSF | | | | CCL1 | | | | ICAM-1 | | | |
| D | IFN-γ | | | | IL-1α | | | | IL-1β | | | |
| E | IL-1 RA | | | | IL-2 | | | | IL-4 | | | |
| F | IL-5 | | | | IL-6 | | | | IL-6R | | | |
| G | IL-7 | | | | CXCL8 | | | | IL-10 | | | |
| H | IL-11 | | | | IL-12 p40 | | | | IL-12 p70 | | | |
| I | IL-13 | | | | IL-15 | | | | IL-16 | | | |
| J | IL-17A | | | | CCL2 | | | | M-CSF | | | |
| K | CXCL9 | | | | CCL3 | | | | CCL4 | | | |
| L | CCL15 | | | | PDGF-BB | | | | CCL5 | | | |
| M | TIMP-1 | | | | TIMP-2 | | | | TNF-α | | | |
| N | TNF-β | | | | TNFRSF1A | | | | TNFRSF1B | | | |

POS, positive control; CXCL13, C-X-C motif ligand 13; CCL11, C-C motif ligand 11; GCSF, granulocyte colony-stimulating factor; GM-CSF, granulocyte macrophage colony-stimulating factor; ICAM-1, intercellular adhesion molecule-1; IFN-γ, interferon-gamma; IL-1α, interleukin-1 alpha; IL-1 RA, interleukin-1 receptor antagonist; M-CSF, macrophage colony-stimulating factor; PDGF-BB, platelet-derived growth factor BB; TIMP-1, tissue inhibitor of metalloproteinases 1; TNF-α, tumor necrosis factor alpha; TNFRSF1A, tumor necrosis factor receptor superfamily member 1A
